# Magnetic resonance imaging pattern recognition of metabolic and neurodegenerative encephalopathies in dogs and cats

**DOI:** 10.3389/fvets.2024.1390971

**Published:** 2024-07-30

**Authors:** María Miguel-Garcés, Rita Gonçalves, Rodrigo Quintana, Patricia Álvarez, Katrin M. Beckmann, Emili Alcoverro, Melania Moioli, Edward J. Ives, Megan Madden, Sergio A. Gomes, Evelyn Galban, Tim Bentley, Koen M. Santifort, An Vanhaesebrouck, Chiara Briola, Patricia Montoliu, Unai Ibaseta, Inés Carrera

**Affiliations:** ^1^Diagnostic Imaging Department, Southern Counties Veterinary Specialists, Independent Vetcare (IVC) Evidensia, Ringwood, United Kingdom; ^2^Department of Small Animal Clinical Science, Small Animal Teaching Hospital, University of Liverpool, Neston, United Kingdom; ^3^Small Animal Hospital, School of Biodiversity, One Health and Veterinary Medicine, University of Glasgow, Glasgow, United Kingdom; ^4^Neurology and Neurosurgery Department, Pride Veterinary Referrals, Independent Vetcare (IVC) Ltd., Derby, United Kingdom; ^5^Section of Neurology, Department of Small Animals, Vetsuisse Faculty Zurich, University of Zurich, Zurich, Switzerland; ^6^ChesterGates Veterinary Specialists, Chester, United Kingdom; ^7^Division of Clinical Radiology, Department of Clinical Veterinary Medicine, Vetsuisse Faculty, University of Bern, Bern, Switzerland; ^8^Anderson Moores Veterinary Specialists, Linnaeus Veterinary Ltd., Hursley, United Kingdom; ^9^Royal (Dick) School of Veterinary Studies, University of Edinburgh, Edinburgh, United Kingdom; ^10^Dovecote Veterinary Hospital, Castle Donington, United Kingdom; ^11^Neurology and Neurosurgery Department, University of Pennsylvania School of Veterinary Medicine, Philadelphia, PA, United States; ^12^IVC Evidensia Small Animal Referral Hospital Arnhem, Neurology, Arnhem, Netherlands; ^13^IVC Evidensia Small Animal Referral Hospital Hart van Brabant, Neurology, Waalwijk, Netherlands; ^14^Queen’s Veterinary School Hospital, Veterinary Department, University of Cambridge, Cambridge, United Kingdom; ^15^Diagnostic Imaging Service, The Ralph Veterinary Referral Centre, Marlow, United Kingdom; ^16^VetCT, Cambridge, United Kingdom; ^17^Anicura Ars Veterinaria Hospital Veterinari, Barcelona, Spain; ^18^Hospital Veterinari Costa Brava, Girona, Spain; ^19^Neurology and Neurosurgery Department, Hospital Veterinario Menes, Gijón, Asturias, Spain; ^20^VetOracle, Norfolk, United Kingdom

**Keywords:** metabolic encephalopathies, neurodegenerative encephalopathies, magnetic resonance imaging, grey matter, white matter, MRI recognition pattern

## Abstract

Metabolic/neurodegenerative encephalopathies encompass a wide list of conditions that share similar clinical and magnetic resonance imaging (MRI) characteristics, challenging the diagnostic process and resulting in numerous tests performed in order to reach a definitive diagnosis. The aims of this multicentric, retrospective and descriptive study are: (I) to describe the MRI features of dogs and cats with metabolic/neurodegenerative encephalopathies; (II) to attempt an MRI recognition pattern classifying these conditions according to the involvement of grey matter, white matter or both; and (III) to correlate the MRI findings with previous literature. A total of 100 cases were recruited, comprising 81 dogs and 19 cats. These included hepatic encephalopathy (20 dogs and three cats), myelinolysis (five dogs), intoxications (seven dogs and one cat), thiamine deficiency (two dogs and seven cats), hypertensive encephalopathy (three dogs and two cats), neuronal ceroid lipofuscinosis (11 dogs and one cat), gangliosidosis (three dogs and two cats), fucosidosis (one dog), L-2-hydroxyglutaric aciduria (13 dogs and one cat), Lafora disease (11 dogs), spongiform leukoencephalomyelopathy (one dog) and cerebellar cortical degeneration (four dogs and two cats). None of the hepatic encephalopathies showed the previously described T1-weighted hyperintensity of the lentiform nuclei. Instead, there was involvement of the cerebellar nuclei (8/23), which is a feature not previously described. Dogs with myelinolysis showed novel involvement of a specific grey matter structure, the claustrum (5/5). Thiamine deficiency affected numerous deep grey nuclei with novel involvement of the oculomotor nuclei (3/9), thalamic nuclei, subthalamus and cerebellar nuclei (1/9). Cats with hypertensive encephalopathy had a more extensive distribution of the white matter changes when compared to dogs, extending from the parietal and occipital lobes into the frontal lobes with associated mass effect and increased brain volume. Lysosomal storage disease showed white matter involvement only, with neuronal ceroid lipofuscinosis characterised by severe brain atrophy when compared to gangliosidosis and fucosidosis. All patients with L-2-hydroxyglutaric aciduria had a characteristic T2-weighted hyperintense swelling of the cerebral and cerebellar cortical grey matter, resulting in increased brain volume. Lafora disease cases showed either normal brain morphology (5/11) or mild brain atrophy (6/11). Dogs with cerebellar cortical degeneration had more marked cerebellar atrophy when compared to cats. This study shows the important role of MRI in distinguishing different metabolic/neurodegenerative encephalopathies according to specific imaging characteristics.

## Introduction

Metabolic and neurodegenerative encephalopathies are uncommon conditions that manifest secondary to derangements of a well-balanced environment encompassing metabolic substrates, neurotransmitters, electrolytes, physiologic pH levels, and blood flow, either by endogenous malfunctions or exogenous toxic effects (1). The degree and type of metabolic product accumulation, the specificity of cell type, anatomical localization of neuronal involvement, and maturation level of the brain at the time of insult determine the nature of the neuropathological changes (2). This complexity results in a wide spectrum of heterogeneous disorders in which various classifications are proposed. Metabolic diseases can be divided according to the aetiology, stratified into acquired or hereditary conditions (Table 1). Acquired disorders can be secondary to endogenous malfunctions or exogeneous toxins, whether hereditary conditions result from a deficit on a gene coding for enzymes directly involved in the affected metabolic pathway (3, 4). Metabolic diseases can also be classified according to the involvement of the grey and white matter (5). Primary encephalopathies affecting the white matter, termed leukodystrophies, are those that affect the integrity of axons and/or their surrounding myelin in the brain. Diseases affecting primarily the grey matter include those involving the susceptible regions of injury at the cortical and deep grey matter structures (such as thalamus and basal nuclei) (5). However, several conditions can affect both grey and white matter to approximately the same extent (1, 6).

**Table 1:** Summary of the most common metabolic/neurodegenerative brain disorders in small animals.

Acquired Endogenous malfunction or toxins Hepatic encephalopathy Kernicterus (bilirubin encephalopathy) Hypoglycemic encephalopathy Myelinolysis/Osmotic demyelination syndrome (e.g., following rapid correction of sodium) Nutritional imbalance: thiamine deficiency, hypocobalaminemic encephalopathy Blood flow disturbance: hypertensive encephalopathy Exogenous toxins (e.g., metronidazole intoxication, bromethalin intoxication, etc.). Hereditary Hereditary neurodegenerative: Lysosomal storage disease Proteinoses: Neuronal ceroid lipofuscinosis. Sphingolipidoses: Globoid cell leukodystrophy, gangliosidosis (GM1 and GM2). Glycoproteinoses: fucosidosis, alpha-mannosidosis, mucopolysaccharidoses (type I and type II). Acidurias: L-2-hydroxyglutaric aciduria Lafora disease Neuroaxonal dystrophies Spongiform leukoencephalomyelopathy (spongy degeneration) Polyencephalopathies 5.6.1 Mitochondrial encephalopathies. 5.6. Leukoencephalomyelopathy of the Rottweiler. Hereditary ataxias Cerebellar cortical degeneration (previously termed cerebellar abiotrophy). Old English sheepdog and Gordon Setter hereditary ataxia associated to *RBA24* genetic variant.

^***^Classification divided into acquired and hereditary conditions.

On the other hand, there is a lot of controversy in the available literature whether to include hypertensive encephalopathy within the category of metabolic conditions. The authors of this study consider that hypertensive encephalopathy meets the definition of metabolic and neurodegenerative encephalopathies previously given by De Oliveira et al. (1), and therefore, the inclusion of this disease in the study is justified.

From a clinical perspective, animals affected by metabolic/neurodegenerative encephalopathies can present with a wide variety of neurological deficits and clinical signs depending on the individual disorder and region(s) of the brain affected. However, the chronic and progressive clinical course, along with the clinical history and signalment (e.g., specific breed), may raise the index of suspicion for a metabolic/neurodegenerative disease.

In magnetic resonance imaging (MRI), they are typically characterised by bilateral, symmetrical intra-axial lesions; however, in many cases, these changes may be non-specific and narrowing down the differential diagnosis is a challenge. In human medicine, there are numerous studies establishing imaging patterns in which patients with different metabolic and neurodegenerative encephalopathies are gathered into groups according to specific imaging features (1, 4, 7–9). Based on this information, diagnostic flow charts can be made in order to guide the clinician during the diagnostic process when interpreting an MRI study.

To date, multiple individual case reports and small case series have described the imaging characteristics of various types of metabolic encephalopathies in veterinary medicine (10–26). However, a large case series comparing the MRI features of metabolic/neurodegenerative diseases with confirmed diagnosis is currently lacking.

This study had the following aims: (I) to describe in detail the MRI features in a large cohort of dogs and cats with confirmed metabolic/neurodegenerative encephalopathies; (II) to attempt a MRI recognition pattern in which the metabolic conditions can be divided according to the anatomical regions involved (grey matter, white matter, and both); and (III) to correlate and compare the MRI findings in the patients included in this study with previous publications available in the literature.

We hypothesised that patients with different metabolic conditions would be able to be classified in different groups according to specific MRI features (e.g., anatomic region involved, involvement of grey or white matter or both). This would allow stratification of the differential diagnosis in these cases, and in some instances, even reach a specific diagnosis when a combination with the clinical history is made.

## Materials and methods

### Study population

In this retrospective multicentric descriptive study, medical records from 17 referral institutions were reviewed to identify dogs and cats with metabolic/neurodegenerative encephalopathies, with a particular focus on the conditions listed in Table 1. Ethical approval was granted by the RCVS Ethics Review Panel. To be included in the study, dogs and cats must have presented to the referral institution with neurological signs and have on the records a complete clinical and neurological examination supporting the suspicion of central nervous system (CNS) disease. Only dogs and cats that had undergone a complete high field MRI study of the brain were selected. For the purpose of this study, a complete MRI series was defined as including a minimum of transverse T2-weighted (T2W), fluid attenuated inversion recovery (FLAIR), and pre-contrast and post-contrast T1-weighted images (T1W). When available, T2*-weighted gradient recalled echo images (T2*) and diffusion-weighted imaging (DWI) and apparent diffusion coefficient (ADC) map were also reviewed. The diagnosis of metabolic/neurodegenerative disease was obtained by confirmation with laboratory or genetic test and/or histopathology, or a combination of the clinical history and improvement after specific treatment implementation. Patients were excluded if the MRI study and clinical data were incomplete, if presented with abnormal cerebrospinal fluid (CSF) analysis suggestive of CNS inflammatory/infectious or neoplastic disease, or if there was presence of other lesions in MRI that could be consistent with an alternative diagnosis (e.g., neoplasia, meningoencephalitis, infarcts or vascular anomalies).

### Clinical features

The following medical record data were collected: species information (dog or cat), age, breed, sex, weight, clinical history, physical examination, neurological examination, previous treatments, blood haematology and biochemistry results, results of the confirmatory test for the specific metabolic/neurodegenerative disease. In addition, treatment after diagnosis, survival time and cause of death were included when available.

Neurological findings that were documented include presence of seizures (none/isolated/cluster seizures/status epilepticus), mentation (normal/obtunded/stupor/coma), behaviour (normal, altered), posture (normal/head tilt/head turn/opisthotonos/cervical ventroflexion), gait (normal, ataxia, and paresis/plegia) and limb(s) affected, proprioception (normal/deficits), vision (normal/unilateral or bilateral deficits), presence of cranial nerve abnormalities (normal/absent menace response, palpebral reflex, pupillary light response, facial sensation, gag reflex and strabismus) and side(s) affected, hyperesthesia (yes/no), and further vestibular anomalies (none, vestibular ataxia, head tilt and nystagmus).

Cerebrospinal fluid analysis, infectious disease testing and urine analysis were also recorded when available.

### Image acquisition and image review

Magnetic resonance imaging protocols and sequences varied between institutions, but all MRI examinations were completed with patients under general anaesthesia, using high-field-strength magnets: 1.5 Tesla (Signa, GE healthcare, Chicago, Illinois, United States; Magnetom Essenza and Amira, Siemens Healthineers, Erlangen, Germany; Toshiba Vantage Elan, Toshiba, Tokyo, Japan; Philips Ingenia Ambition, Philips, Amsterdam, the Netherlands). Anaesthetic protocols were varied and adapted to individual patients by the attending anaesthetist. The MRI studies were reviewed by a European College of Veterinary Diagnostic Imaging board-certified veterinary radiologist (IC), and a second-year veterinary radiology resident (MM-G) on DICOM viewing software (OsiriX Imaging Software, 12.0 MD, Pixmeo, Geneva, Switzerland). Reviewers were aware of the history, patient signalment, clinical, and neurological examination findings. The MRI studies were evaluated individually and in consensual evaluation.

**Figure 1:**
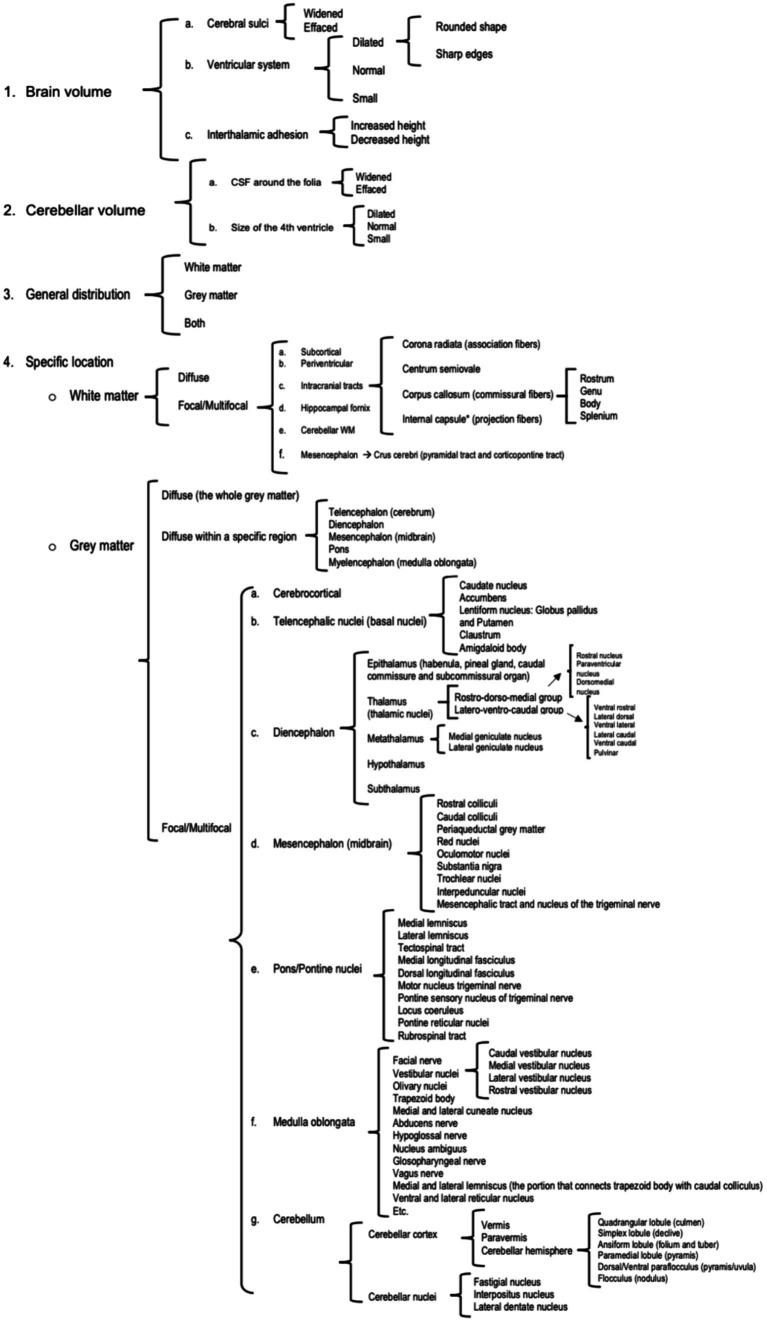
Anatomic map designed to guide the assessors during the imaging evaluation.

Prior to the image review, an anatomical map was designed based on previously published veterinary neuroanatomy textbooks (27, 28) in order to guide the evaluation of the studies in a methodical manner (Figure 1). The specific MRI features assessed are summarised in Table 2.

**Table 2:** Specific MRI features evaluated during groups classification.

MRI features	Types	Evaluation method
Forebrain volume	Decreased	Subjectively defined as volume loss leading to enlargement of the ventricles and subarachnoid spaces, and decreased interthalamic height. Grading of the atrophy was performed subjectively ranging from mild to severe.
	Normal	
	Increased	Increased brain volume or brain swelling was considered when there was loss of the normal T2W hyperintense signal from the CSF within the cerebral sulci; and/or when presence of transtentorial and cerebellar herniation.
Cerebellar volume	Decreased	Atrophy was defined subjectively as volume loss leading to enlargement of the fourth ventricle and increase conspicuity of the CSF between the cerebellar folia. Grading of the atrophy was performed subjectively ranging from mild to severe.
	Normal	
	Increased	Increased cerebellar volume was considered when loss of the CSF signal within the cerebellar sulci, decreased size of the fourth ventricle and/or when there was the presence of cerebellar herniation or caudal displacement of the uvula.
Grey/white matter definition	Decreased	This was evaluated in general, but as reference the parietal lobes and basal nuclei were specifically noted.
	Normal	
	Increased	
Anatomical location	Grey matter	Detailed description of the anatomical location of the lesions within either the grey matter, white matter or both based on previously published veterinary neuroanatomy textbooks (27, 28).
	White matter	
	Both	
Distribution	Diffuse	Distribution was classified as diffuse when extensive involvement of the white or grey matter.
	Focal/multifocal	Distribution was classified as focal/multifocal when affecting a specific anatomical region.
Lesion margination	Well-defined	Lesion margination was characterised subjectively as well or poorly circumscribed.
	Ill-defined	
Signal intensity	Hyperintense	Description of the signal intensity pattern of the lesions in all sequences compared to normal white matter (T2W, FLAIR, and T1W pre- and post-contrast).
	Isointense	
	Hypointense	
Contrast enhancement		Evidence of contrast enhancement (absent, mild, moderate, and severe) and its pattern (homogeneous, heterogeneous, and rim-like enhancement) were evaluated.
Meningeal enhancement	Pachymeningeal	Enhancement of the dura mater without extension into the cerebral sulci.
	Leptomeningeal	Enhancement of the arachnoid and pia mater which extends into the sulci.
Lentiform nuclei		The T1W signal intensity of the lentiform nuclei was compared between patients with hepatic encephalopathy to patients with other metabolic conditions.

Following the analysis of the images, patients were grouped according to their subjective brain volume (decreased, increased or normal), and according to the predominant anatomic distribution of the lesions (involving only grey matter, involving only white matter or involving both grey and white matter). For each group classified according to grey/white matter involvement, the specific anatomic regions affected were noted and compared.

## Results

### Signalment, clinical signs and MRI findings

A total of 100 cases, comprising 81 dogs and 19 cats, were recruited for the study. These cases included animals across 15 different final diagnoses: hepatic encephalopathy (20 dogs and three cats), myelinolysis (five dogs), four different exogenous intoxications [metronidazole (five dogs), *Cycas revoluta* (one dog), ethylene glycol (one dog), bromethalin (one cat)], thiamine deficiency (two dogs and seven cats), hypertensive encephalopathy (three dogs and two cats), neuronal ceroid lipofuscinosis (11 dogs and one cat), gangliosidosis (three dogs and two cats), fucosidosis (one dog), L-2-hydroxyglutaric aciduria (13 dogs and one cat), Lafora disease (11 dogs), spongiform leukoencephalomyelopathy (one dog) and cerebellar cortical degeneration (four dogs and two cats). Further information regarding the species, breeds, age, sex status and MRI findings are summarised below for each of these diagnoses. Additional information such as numbers of each breed, neurological signs and diagnosis are summarised for all conditions in Supplementary material A.

#### Endogenous malfunctions and toxins

##### Hepatic encephalopathy

###### Dogs

A total of 20 dogs were included in the study. In two dogs, the cause was the presence of multiple acquired shunts secondary to advanced liver disease (liver cirrhosis), whether the remaining patients presented with congenital portosystemic shunts of variable morphology. Affected canine breeds were Shih Tzu, Border Terrier, Standard Schnauzer, West Highland White Terrier, Cairn Terrier, Chihuahua, English Cocker Spaniel, English Sheepdog, Entlebucher Mountain dog, Lurcher, Miniature Pinscher, Labradoodle, Poodle (toy) and a crossbreed. Median age at the presentation was 5 years old (2.5 months–12 years old). Nine dogs were neutered males, three dogs were intact males, five dogs were female spayed and two females entire.

Regarding the MRI evaluation, no morphological abnormalities were detected in two dogs (2/20). The remaining patients showed abnormalities on MRI (18/20). The most common abnormality was reduced volume of the brain (15/20), which showed variable degrees of forebrain atrophy alone (3/20) or in combination with cerebellar atrophy (12/16). The degrees of atrophy were mild in seven cases and moderate in the other 11 patients. Severe brain atrophy was not seen in any of the cases.

**Figure 2:**
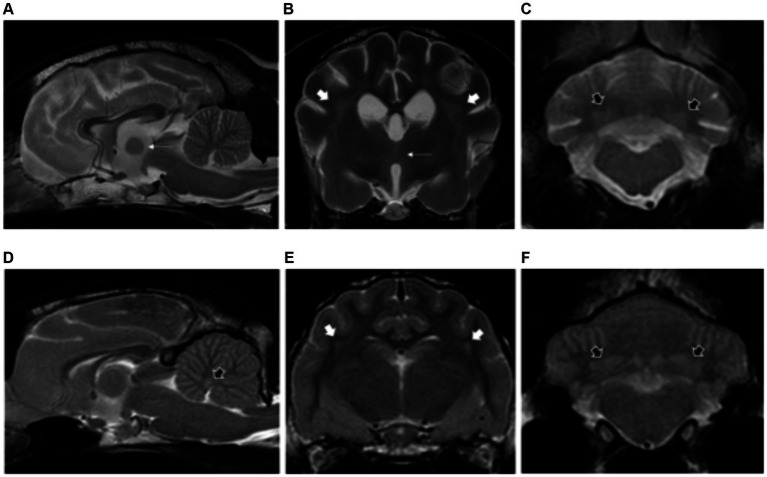
Sagittal and transverse plane T2-weighted images at the level of the forebrain and cerebellum of a dog **(A–C)** and cat **(D–F)** diagnosed with hepatic encephalopathy. Both patients have bilateral and symmetric hyperintensities affecting the centrum semiovale and extending towards the corona radiata (short white arrows) **(B,E)** as well as at the cerebellar nuclei (short black arrows) **(C,D,F)**. Note also the moderate brain atrophy in the canine patient evidenced by the decreased interthalamic height (long white arrows) and widening of the cerebral and cerebellar sulci **(A–C)**.

A total of 13 patients presented bilateral and symmetric intra-axial lesions affecting both the white and grey matter. Within the white matter, the centrum semiovale and corona radiata were typically affected (10/15), with changes at the internal capsule also seen (3/15). Within the grey matter, the cerebellar nuclei were affected in 6/13 patients (including fastigial, interpositus and lateral dentate nuclei). The lesions were all homogenous, T2W and FLAIR hyperintense, T1W isointense to white matter and with no evidence of contrast enhancement (Figures 2A–C).

###### Cats

Three cats with congenital portosystemic shunts were included in the study. Affected feline breeds included domestic longhair, British Shorthair and Sphynx. Median ages at presentation was 6 years old (range: 4–8 years old). Two cats were neutered males and one cat was a female spayed.

Magnetic resonance imaging evaluation showed normal brain volume (2/3) or evidence of mild forebrain atrophy (1/3). Similar to dogs, changes of the white matter were most common at the centrum semiovale and corona radiata (2/3), with changes also visible at the internal capsule (1/3). The grey matter was also affected, including the cerebellar nuclei (2/3) as described in dogs, and also the medial longitudinal fasciculus and reticular formation (1/3) (Figures 2D–F).

##### Myelinolysis/osmotic demyelination syndrome

Five dogs consisted of English Cocker Spaniel, Lhasa Apso, Standar Schnauzer and crossbreed, met the inclusion criteria. Median age at presentation was 5.5 years (range: 6 months–15 years old). Three dogs were spayed female dogs, one dog male neutered and one dog male entire.

**Figure 3:**
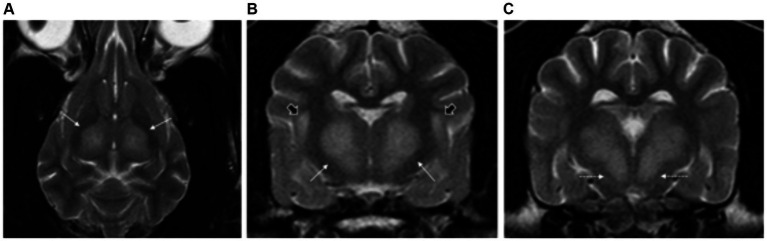
Dorsal **(A)** and transverse plane T2-weighted images at the level of the thalamus **(B,C)** of a dog with presumptive diagnosis of osmotic demyelination syndrome. There are bilateral and symmetric hyperintensities affecting the thalamus (long white arrows) **(A–C)** and extending into the subthalamus (dashed long white arrows) **(C)**. A specific region of the grey matter is also affected, corresponding with the claustrum (short black arrows) **(B)**.

>The brain volume was normal in all cases (5/5). Patients showed grey matter structures affected with the presence of bilateral, symmetric intra-axial lesions. The grey matter structures affected included thalamus (5/5), subthalamus (1/5) and a specific anatomical region located immediately lateral to the internal capsule (5/5), corresponding with the claustrum (Figure 3).

##### Nutritional imbalance–thiamine deficiency

###### Dogs

Two dogs with thiamine deficiency met the inclusion criteria. Both crossbreed dogs, a female spayed and male neutered, with a median age of 8 years.

**Figure 4:**
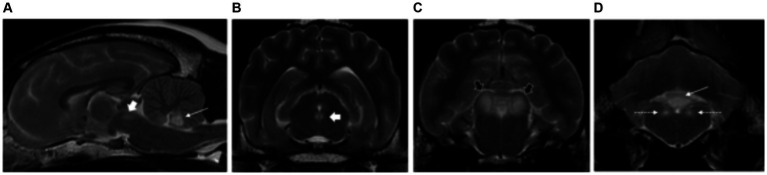
Sagittal **(A)** and transverse plane T2-weighted images of a dog with thiamine deficiency **(B–D)**. Hyperintense lesions are noted affecting the oculomotor nuclei (short white arrows) **(A,B)**; cerebellar nodulus (long white arrows) **(A,D)**; caudal colliculi (black arrows) **(C)**; and vestibular nuclei (dashed white long arrows) **(D)**.

All patients had normal forebrain and cerebellar volume (2/2). Bilateral, symmetric intra-axial lesions were seen affecting the grey matter of the oculomotor nuclei (2/2), caudal colliculi (2/2), red nuclei (1/2), vestibular nuclei (1/2) and cerebellar nodulus (1/2). These lesions were T2W and FLAIR hyperintense, T1W isointense to grey matter with mild contrast enhancement (Figure 4).

###### Cats

Seven cats consisted of domestic shorthair, British Shorthair and Ragdoll, were included in the study. The median age was 5 years (range of 1–8 years). Three cats were female entire, another three were female neutered and one cat was male castrated.

**Figure 5:**
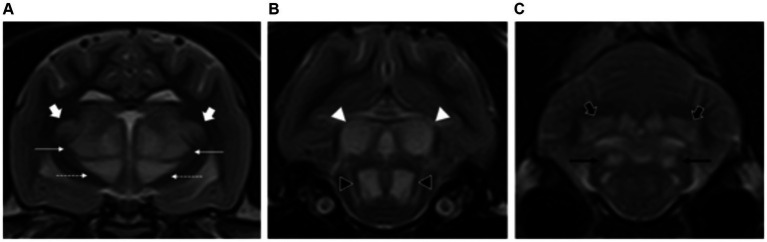
Transverse plane T2-weighted images of a cat with thiamine deficiency at the level of the thalamus **(A)**, midbrain **(B)**, and cerebellum **(C)**. There are bilateral and symmetric hyperintensities affecting multiple deep grey matter nuclei: lateral geniculate (short white arrows), thalamus (long white arrows), and subthalamus (dashed long white arrow) **(A)**; caudal colliculi (white arrowhead) and oculomotor nuclei (black arrowhead) **(B)**; and cerebellar (short black arrows) and vestibular nuclei (long black arrows) **(C)**.

All patients had normal forebrain and cerebellar volume (7/7). Bilateral, symmetric intra-axial lesions were seen affecting the deep grey matter structures in all cases. The locations included: lateral geniculate nuclei (7/7), vestibular nuclei (6/7), facial nuclei (5/7), caudal colliculi (5/7), cerebellar nodulus (4/7), red nuclei (1/7), rostral colliculi (1/7) and periaqueductal grey matter (1/7). Additional anatomical regions were the oculomotor nuclei (1/7), thalamic nuclei (1/7), subthalamus (1/7) and cerebellar nuclei (1/7). All the intra-axial lesions were T2W and FLAIR hyperintense, T1W isointense to grey matter and some of them showed mild contrast enhancement (3/7) (Figure 5). Three patients presenting with seizures showed additional findings affecting the cortical grey matter of the forebrain at the parietal lobes (1/3), occipital lobes (1/3), cingulate gyrus (1/3) and hippocampus (2/3). These changes were characterised by bilateral symmetric T2W and FLAIR hyperintensities, T1W isointense to grey matter with evidence of contrast enhancement in one case.

##### Blood flow disturbance–hypertensive encephalopathy

###### Dogs

Three dogs consisted of Bedlington Terrier, Dalmatian and a crossbreed, were included in the study. The median age was 12.5 years of age (range of 11–14 years of age). One dog was a castrated male whether the other two were female spayed dogs.

**Figure 6:**
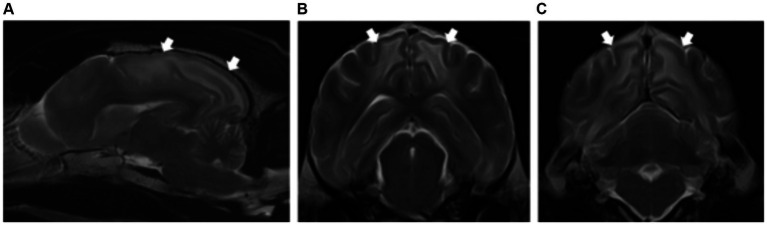
Left parasagittal **(A)** and transverse T2-weighted images at the level of the parietal **(B)** and occipital lobes **(C)** of a dog with hypertensive encephalopathy. Note the bilateral and symmetric hyperintensities of the white matter (short white arrows). Contrary to the feline patient, the changes in this dog do not extend towards the frontal region and there is no associated mass effect.

On MRI evaluation, there were bilateral and symmetric intra-axial lesions affecting the white matter of the forebrain, more marked from the parietal to occipital lobes, and especially at the internal capsule and corona radiata (3/3). Involvement of the grey matter was also seen with the caudate nuclei affected in one dog. None of these changes were associated to mass effect and the brain volume was judged as normal. The lesions were homogeneous, ill-defined, hyperintense in T2W and FLAIR, isointense in T1W and without contrast enhancement. DWI and ADC map were available in two cases, showing no evidence of restricted diffusion, being hyperintense on both images (Figure 6).

###### Cats

Two cats were included in the study. A female entire Abyssinian cat and one male neutered domestic shorthair of 15 and 3 years old, respectively.

**Figure 7:**
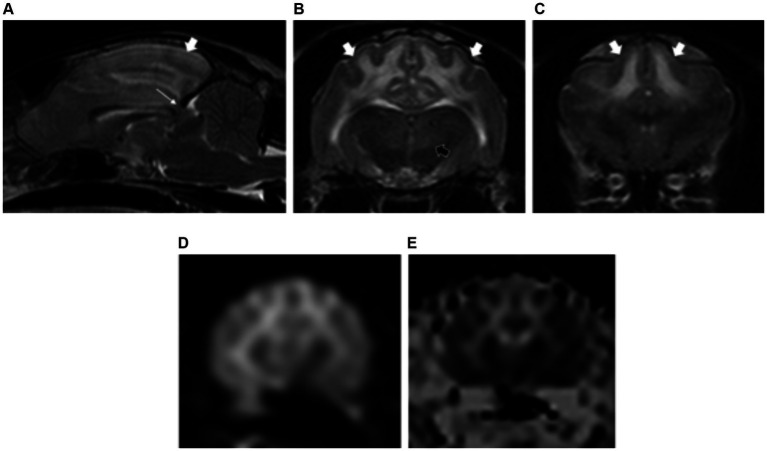
Sagittal T2-weighted image **(A)** of a cat with hypertensive encephalopathy. Note the associated mass effect with caudal displacement of the midbrain across the tentorium cerebelli (caudal transtentorial herniation) (long white arrow). Transverse plane T2-weighted images showing the white matter hyperintensities (short white arrows) extending from the occipital and parietal lobes **(A,B)** to the frontal lobes **(C)**. Bilateral and symmetric hyperintensities are also noted affecting the thalamus (short black arrow) **(B)**. Diffusion-weighted **(D)** and apparent diffusion coefficient (ADC) map images **(E)** showing facilitated diffusion of the white matter lesions, confirming the vasogenic origin of the oedema.

Feline patients had a more dramatic involvement of the white matter with a diffuse involvement of the corona radiata from the frontal to occipital lobes (2/2). Involvement of the grey matter of the thalamus was also seen in one cat. There was associated mass effect and increased brain volume with evidence of caudal transtentorial herniation in both cases and marked ventral displacement of the thalamus in one case. The lesions were ill-defined, homogeneous, hyperintense in T2W and FLAIR, isointense in T1W and without contrast enhancement. The lesions were hyperintense in DWI and ADC map, indicating facilitated diffusion (2/2). (Figures 7).

#### Exogeneous toxins

A total of eight patients suffered from exogenous intoxication. These included five dogs with metronidazole intoxication, one dog with *Cycas revoluta* intoxication, one dog with ethylene glycol intoxication and one cat with bromethalin intoxication.

Canine breeds included Labrador Retriever, German Shepherd, Golden Retriever, Jack Russell Terrier, Northern Inuit and Siberian Husky. The median age was 6 years (range of 8 months–11 years of age). Two dogs were spayed female dogs, two dogs female entire, two dogs were male neutered and another dog male entire.

The cat with bromethalin intoxication was a 3-year-old, female neutered, domestic shorthair.

**Figure 8:**
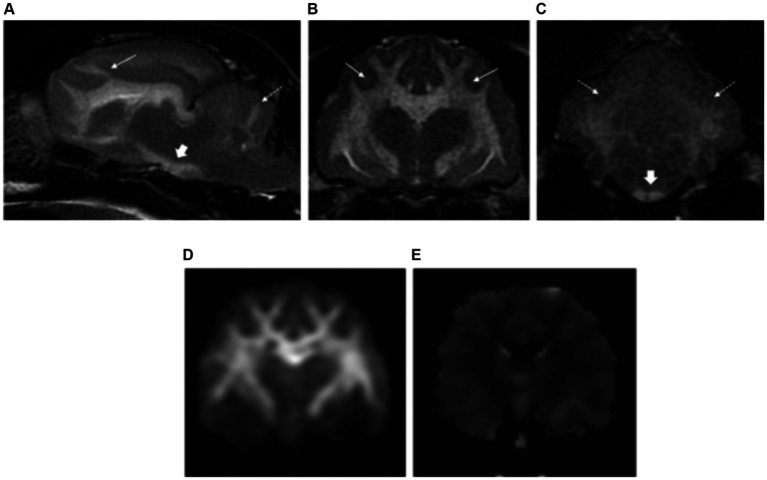
Left parasagittal **(A)** and transverse plane T2-weighted images at the level of the forebrain **(B)** and cerebellum **(C)** of a cat with bromethalin intoxication. There is severe brain swelling with effacement of the normal cerebral and cerebellar sulci diffusely. Severe diffuse hyperintensities are visible along the white matter of the forebrain (long white arrows), cerebellum (dashed long white arrows) and along the crus cerebri towards the pyramidal tracts of the medulla oblongata (short white arrows). Diffusion-weighted **(D)** and apparent diffusion coefficient (ADC) map images **(E)** showed restricted diffusion of the white matter lesions, confirming the cytotoxic origin of the oedema.

All cases of metronidazole intoxication had normal brain volume (5/5). Two patients showed bilateral symmetric lesions affecting the grey matter, specifically the red nuclei (2/2), vestibular nuclei (2/2) and cerebellar nuclei (1/2). The remaining intoxications showed evidence of increased forebrain and cerebellar volume (3/8). Additionally, all cases had predominant involvement of white matter structures including: cerebellar white matter (2/3), diffuse involvement of white matter of the forebrain more marked at corona radiata (3/3) and internal capsule (2/3); corpus callosum (1/3) and along the crus cerebri to the pyramidal tracts of the medulla oblongata (2/3). Two cases (*Cycas revoluta* and ethylene glycol intoxications) had also partial involvement of grey matter (thalamus). The lesions were homogeneous, markedly hyperintense on T2W and FLAIR images. In two cases, DWI/ADC map were available, revealing restricted diffusion (hyperintense on DWI and hypointense on ADC map) (Figure 8).

#### Hereditary neurodegenerative

##### Lysosomal storage disease

###### Neuronal ceroid lipofuscinosis

####### Dogs

A total of 11 dogs consisted of Chihuahua, Field Spaniel, Jack Russell, Tibetan Terrier and crossbreed, were included in the study. The median age was 2.5 years (range of 1.5–5 years). Six dogs were castrated males, one dog was entire male and four were female spayed.

**Figure 9:**
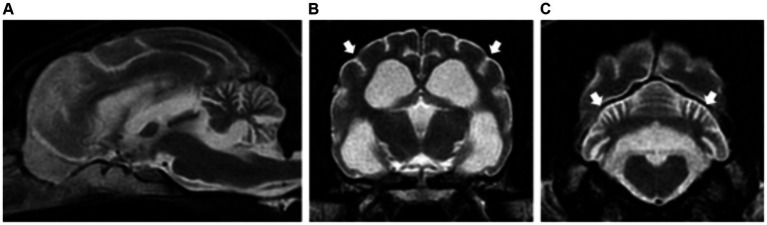
Sagittal **(A)** and transverse plane T2-weighted images of a dog with neuronal ceroid lipofuscinosis (**B,C**). Note the severe degree of brain and cerebellar atrophy with widening of the cerebral and cerebellar sulci (short white arrows). There is associated thinning of the cortical grey matter as well as diffuse T2W hyperintensity of the white matter resulting in a decreased grey matter/white matter definition.

On MRI all dogs showed a characteristic severe atrophy of the forebrain and cerebellum (11/11). There was associated thinning of the cortical grey matter as well as diffuse T2W and FLAIR hyperintensity of the white matter resulting in a decreased grey matter/white matter definition (11/11). The corpus callosum show also increase T2W hyperintensity, becoming isointense to the grey matter in six dogs (Figure 9). Two patients had diffuse pachymeningeal enhancement on the post-contrast images. One dog had large accumulation of fluid within the subdural space of the forebrain bilaterally, being T2W hyperintense, T1W mildly hyperintense to CSF and not supressing on FLAIR. On T2* images, there was irregular signal void in this region, indicating the presence of blood degradation products. A chronic subdural haematoma was confirmed on histopathology.

####### Cats

A 2-year-old, male neutered, domestic shorthair cat met the inclusion criteria.

Similar to dogs, MRI showed severe atrophy of the forebrain and cerebellum with thinning of the cortical grey matter, as well as diffuse and mild T2W and FLAIR hyperintensity of the white matter.

###### GM1 and GM2 gangliosidosis

####### Dogs

Three dogs met the inclusion criteria. These included two Shiba Inu with GM2 gangliosidosis and a crossbreed with GM1 gangliosidosis. They were two females neutered and one male neutered, with a median age of 1.4 years (14 months–18 months old).

**Figure 10:**
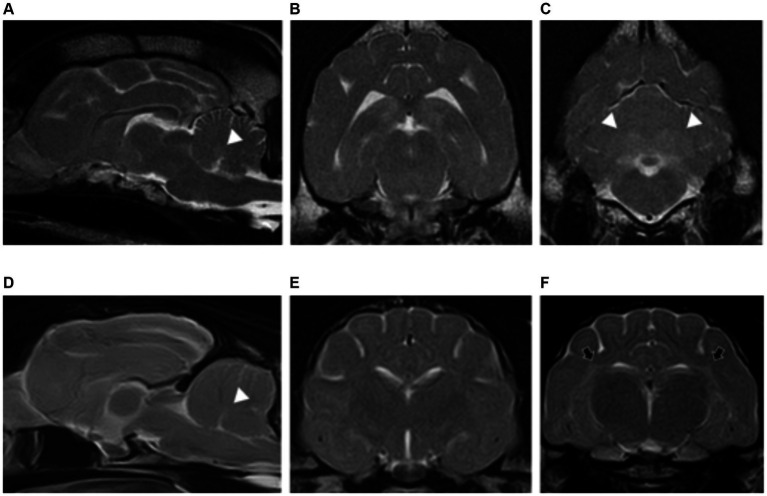
Sagittal **(A)** and transverse plane T2-weighted images at the level of the forebrain **(B)** and cerebellum **(C)** of a dog with GM2 gangliosidosis. Sagittal **(D)** and transverse T2-weighted of the forebrain **(E,F)** of a cat with GM1 gangliosidosis. Similar findings are visible for both patients. There is diffuse hyperintensity of the white matter of the forebrain with subsequent decrease of the normal grey matter/white matter definition. In the cat, this is especially marked at the corona radiata (short black arrows) **(F)**. The changes are also visible affecting the cerebellar white matter of both patients (white arrowheads) **(A,C,D)**.

One dog had mild forebrain and cerebellar atrophy. The remaining of the patients had normal brain volume (2/3). All patients had similar MRI features with diffuse involvement of white matter, homogeneously hyperintense in T2W and FLAIR, resulting in decreased on the normal grey matter/white matter distinction (as white matter became isointense to grey matter). The white matter hyperintensities were more marked at the level of the internal capsule and corona radiata in all cases (3/3), but also along the corpus callosum, crus cerebri and pyramidal tracts (2/3). These changes were isointense to the white matter on T1W images with no evidence of post-contrast enhancement (Figures 10A–C).

####### Cats

Two domestic shorthair breed cats with GM1 gangliosidosis met the inclusion criteria. One male and one female, both neutered. The median age was 8 months (range of 4 months–12 months).

Feline patients had similar MRI features as described in dogs, with diffuse T2W and FLAIR hyperintense white matter of the forebrain and cerebellum causing decreased on the normal grey matter/white matter distinction. These findings were more marked at the level of the internal capsule and corona radiata in all cases. The symmetrical diffuse intra-axial white matter lesions were isointense on T1W images with no evidence of contrast enhancement (Figures 10D–F).

###### Fucosidosis

Only one case was included in the study being a female, neutered, Springer Spaniel of 2 years of age.

**Figure 11:**
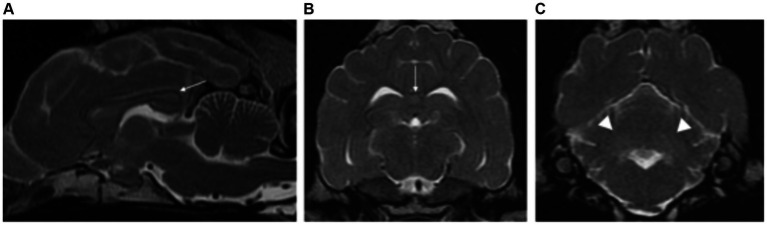
Sagittal **(A)** and transverse plane T2-weighted images of the forebrain **(B)** and cerebellum **(C)** of a dog with fucosidosis. There is diffuse hyperintensity of the white matter of the forebrain with subsequent decrease in the normal grey/white matter distinction. This is especially marked at the corpus callosum (long white arrows) **(A,B)**. The cerebellar white matter is also affected (white arrowheads) **(C)**.

This dog had similar MRI findings than those diagnosed with GM1 and GM2 gangliosidosis, characterised by diffuse involvement of forebrain and cerebellar white matter, T2W and FLAIR hyperintense, with subsequent decrease in the normal grey matter/white matter definition. These changes were more marked at the corona radiata and corpus callosum. On T1W images they were isointense with no evidence of contrast enhancement. (Figure 11).

##### L-2-hydroxyglutaric aciduria

###### Dogs

Thirteen dogs consisted of Staffordshire Bull Terrier, Yorkshire, West Highland White Terrier and crossbreed, met the inclusion criteria. Median age at presentation was 3 years (range between 4 months and 6 years old). Four dogs were male neutered, two dogs male entire, four dogs were female spayed and three dogs female entire.

**Figure 12:**
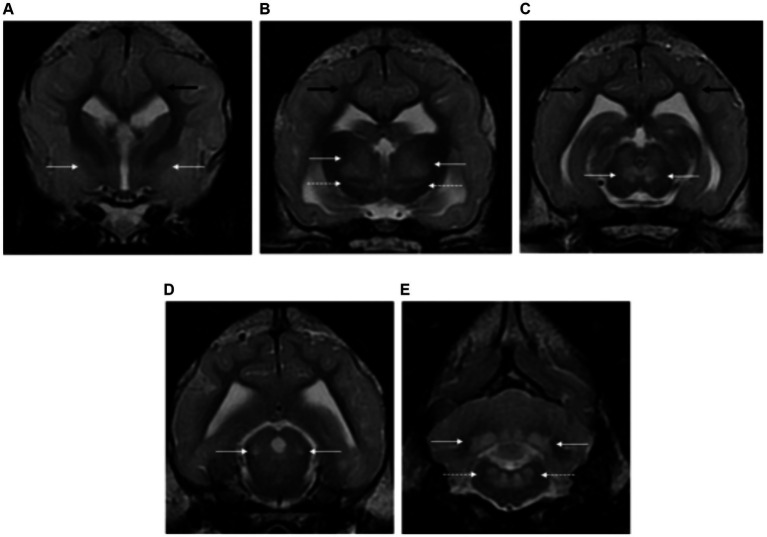
Transverse plane T2-weighted images of the brain of a dog with L-2-hydroxyglutaric aciduria. Note the diffuse cortical grey matter swelling of the forebrain and cerebellum with increased signal intensity **(A–E)**. There are bilateral and symmetrical lesions affecting the globus pallidus and putamen (long white arrows) **(A)**, thalamus (long white arrow) and subthalamus (dashed long white arrows) **(B)**, oculomotor nuclei (long white arrows) **(C)**, lateral lemniscus (long white arrows) **(D)** and cerebellar nuclei (long white arrows) and vestibular nuclei (dashed long white arrows) **(E)**. The subcortical white matter is also mildly affected (long black arrows).

All patients showed an increased forebrain and cerebellar volume secondary to a thickened cortical grey matter (13/13). The cortical grey matter was diffusely and homogeneously T2W and FLAIR hyperintense. In addition, all cases had bilateral symmetric focal intra-axial lesions affecting the grey matter at the following locations: thalamus (13/13), subthalamus (10/13), rostral colliculi (12/13), caudal colliculi (13/13), cerebellar nuclei (11/13), oculomotor (12/13), vestibular nuclei (12/13), red nuclei (9/13), lateral lemniscus (6/13), medial geniculate nuclei (5/13), lateral geniculate nuclei (2/13), globus pallidus and putamen (7/14). The white matter was also mildly affected to a lesser extent (5/14), predominantly at the subcortical white matter of the forebrain and cerebellar white matter surrounding the cerebellar nuclei (Figure 12). All lesions were hyperintense in T2W and FLAIR, isointense in T1W and without contrast enhancement. Only one case included DWI and ADC sequences, and this revealed restricted diffusion (hyperintense in DWI and hypointense in ADC map).

###### Cats

The only cat included was a 7 month-old, female neutered, domestic shorthair.

**Figure 13:**
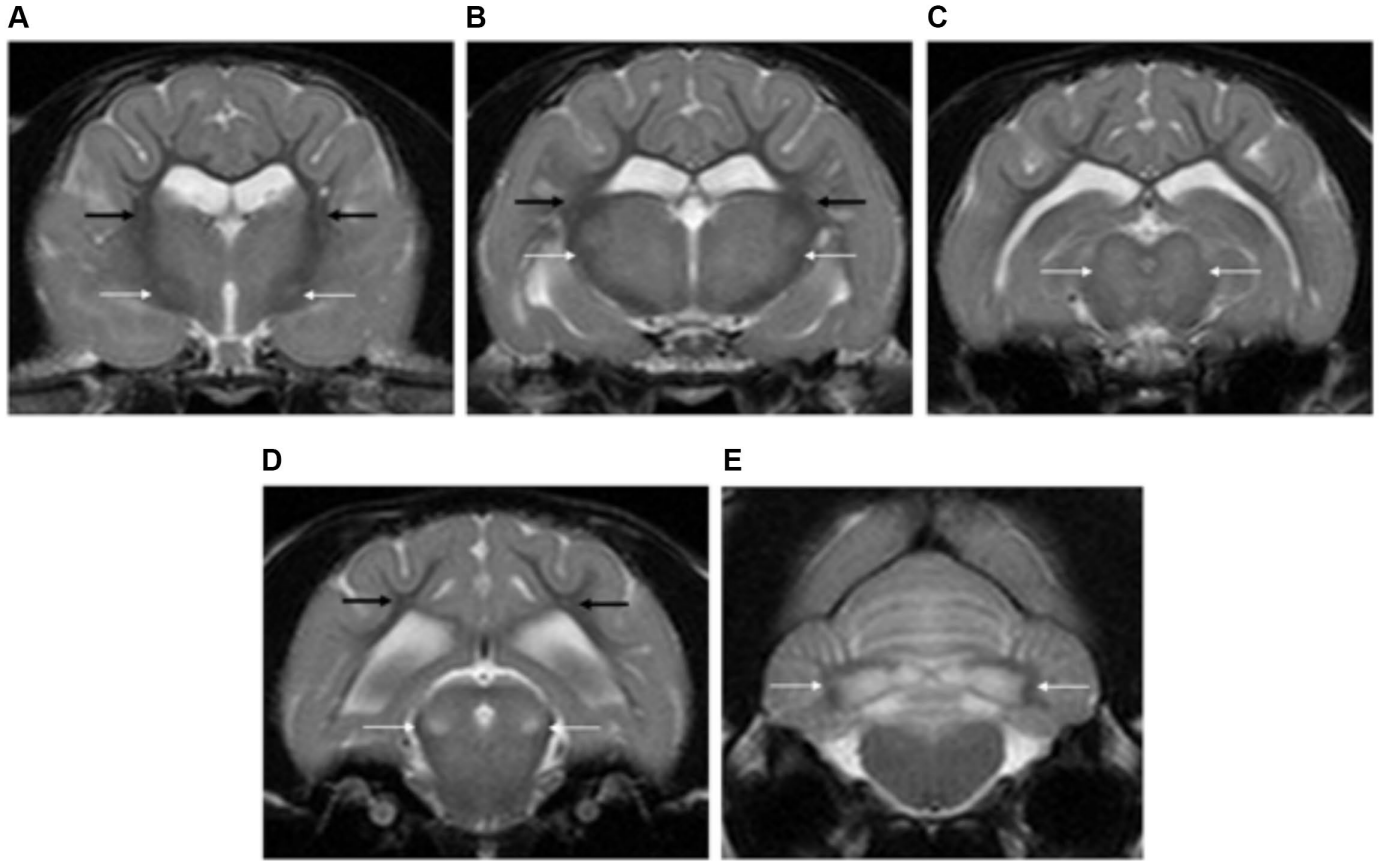
Transverse plane T2-weighted images of the brain of a cat with L-2-hydroxyglutaric aciduria. Note the diffuse cortical grey matter swelling of the forebrain and cerebellum with increased signal intensity **(A–E)**. There are bilateral and symmetrical lesions affecting the globus pallidus and putamen (long white arrows) **(A)**, thalamus (long white arrow) **(B)**, oculomotor nuclei (long white arrows) **(C)**, lateral lemniscus (long white arrows) **(D)** and cerebellar nuclei (long white arrows) **(E)**. The subcortical white matter is also mildly affected (long black arrows).

Magnetic resonance imaging findings included increased forebrain and cerebellar volume secondary to a diffusely swollen cortical grey matter. Bilateral symmetric focal intra-axial lesions were seen affecting the thalamus, subthalamus, oculomotor nuclei, lateral lemniscus, cerebellar nuclei, vestibular nuclei and rostral and caudal colliculi (Figure 13). All lesions were homogeneous, hyperintense in T2W and FLAIR, isointense in T1W and without contrast enhancement. DWI/ADC map was not available in this case.

##### Lafora disease

Eleven dogs consisted of Beagle, Basset hound, Brussels Griffon, Chihuahua, Wire haired Dachshund and crossbreed, met the inclusion criteria. The median age was 8.5 years (range of 6–10 years). They were five male (two intact, three neutered) and six female (one intact, five neutered).

The brain was unremarkable in five cases. Six dogs had mild degree of forebrain and cerebellar atrophy. None of the dogs showed evidence of intra-axial lesions.

##### Spongiform leukoencephalomyelopathy

Only one case met the inclusion criteria, being a female entire Belgian Malinois dog of 5 months old.

The patient had normal brain volume and bilateral symmetrical lesions affecting the cerebellar nuclei. These lesions were T2W and FLAIR hyperintense, T1W isointense and non-contrast enhancing.

##### Cerebellar cortical degeneration

###### Dogs

Four dogs consisted of German Wire Hired Pointer, Jack Russell, Shiba Inu and Staffordshire Bull terrier, were included in the study. The median age was 5 years old (range of 4–6.5 years old). Three dogs were male neutered and one dog male entire.

On MRI, all cases had evidence of cerebellar atrophy, being the remaining intracranial structures within normal limits (4/4).

###### Cats

Two cats met the inclusion criteria. Both patients, one domestic shorthair and one Ragdoll, were 6 months old. One cat was male entire and one female entire.

All cases had cerebellar atrophy, being the remaining brain structures within normal limits (2/2). The degree of cerebellar atrophy was less marked when compared to dogs.

### Group analysis

**Figure 14:**
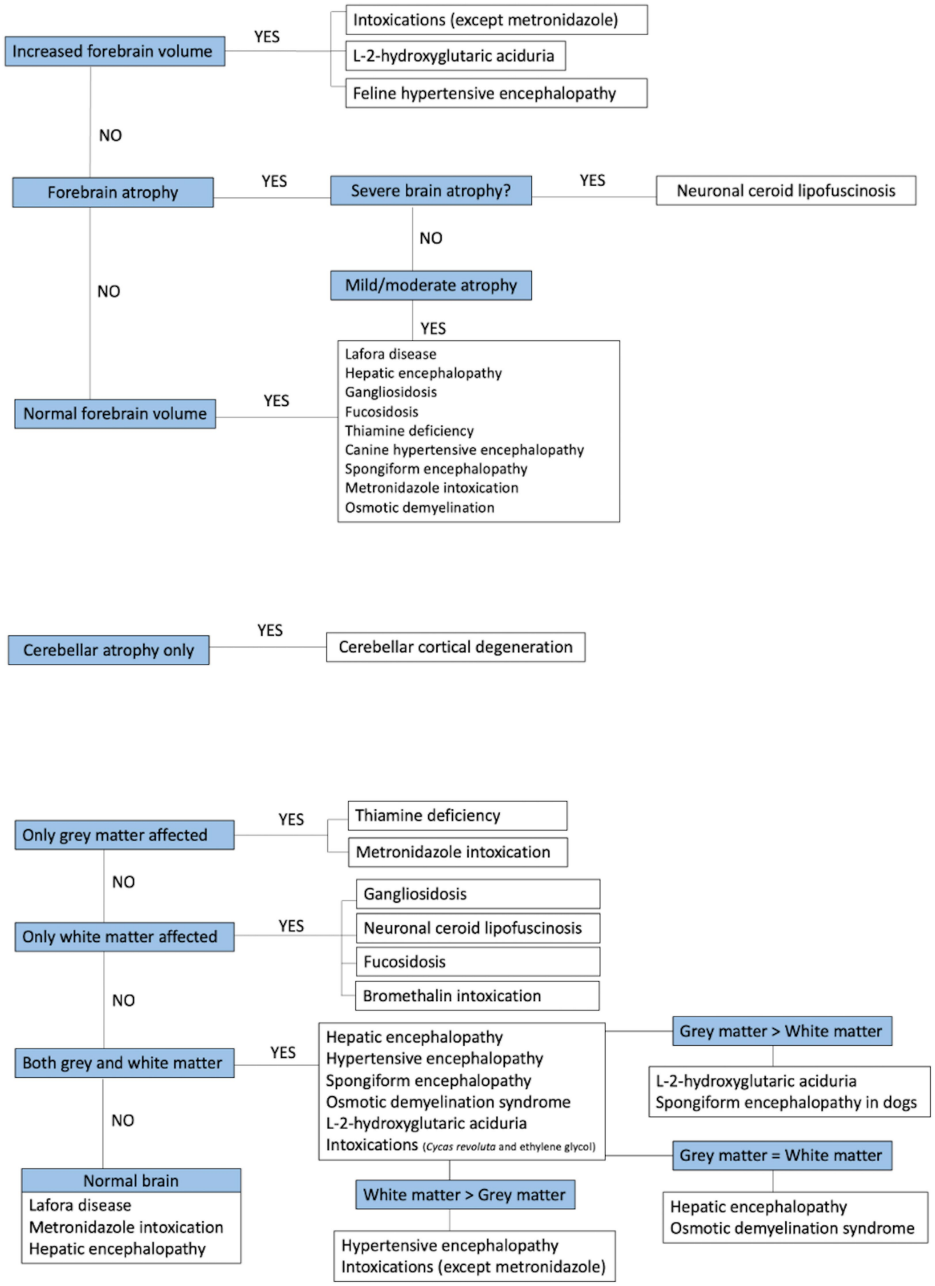
Flow chart summarising the group analysis of metabolic encephalopathies.

Figure 14 illustrates a flow chart, which serves as a further classification of metabolic/neurodegenerative diseases based on the MRI findings on the patients included in this study. This classification includes brain volume (increased, decreased and normal) and distribution of the lesions (grey and/or white matter).

## Discussion

This study demonstrates that it is possible to group patients with different metabolic and neurodegenerative encephalopathies based on specific MRI features, leading to the development of a pattern recognition system to guide the diagnostic process of these conditions by prioritising differential diagnosis.

### Hepatic encephalopathy

Hepatic encephalopathy was the most common metabolic disorder encountered in our study with a total of 23 cases. Similar to what has been previously reported, patients had normal brain morphology on MRI (2/23) or evidence of brain atrophy ranging from mild to moderate (16/23) (18, 29). Fifteen patients showed T2W and FLAIR hyperintensity of the centrum semiovale and internal capsule, which may suggest oedema. Previous reports have described other grey matter structures affected (such as medial longitudinal fasciculus and reticular formation) (19). These findings were also seen in one of our feline patients. Involvement of the cerebellar nuclei was seen in eight patients with hepatic encephalopathy included in this study, a feature that has not been previously described in the literature. The reasons why these grey matter structures are affected are not known. Hyperintensity in T1W images of the lentiform nuclei have been reported to be a feature of hepatic encephalopathy due to manganese deposition (18, 30). However, none of our cases showed abnormal increased of T1W signal intensity of the lentiform nuclei, when compared to the other dogs included in this study. Therefore, this feature should be considered with caution and in light with other more prevalent characteristic findings of hepatic encephalopathy (such as brain atrophy, involvement of the centrum semiovale, internal capsule and cerebellar nuclei). Further detailed discussion about imaging findings and pathophysiology is described in Supplementary material B.

### Osmotic demyelination syndrome

Similar to what has been previously reported, all dogs included in this study with osmotic demyelination syndrome showed bilateral and symmetric lesions affecting the thalamus (5/5) (16, 31). The subthalamus was also affected in one dog, a feature not previously described in the literature (16, 31). In an experimental study where myelinolysis was induced in dogs, additional grey matter regions affected were the pons, caudate nucleus, putamen nucleus and midbrain (32). This is similar to the lesion characteristic and distribution that have been reported in humans (33). The pathophysiology of this condition is further described in Supplementary material B.

A novel finding not previously described was the involvement of a specific grey matter structure seen in all patients, the claustrum. The claustrum is a component of the basal nuclei (27). The reason for the susceptibility of this specific region in these patients is unknown and histopathological correlation is required to confirm the nature of the changes.

### Thiamine deficiency

The nine cases of thiamine deficiency included in our study showed predominant involvement of multiple deep grey matter structures in agreement with the results from previous studies (21, 22). Novel imaging findings not previously reported were the involvement of the oculomotor nuclei in two dogs and one cat, as well as the thalamic nuclei, subthalamus and cerebellar nuclei in one cat. Histologically, the oculomotor nuclei have been previously recognised to be affected in dogs with thiamine deficiency (34). However, no studies have reported the appearance of these changes on MRI. The selective involvement of different brain areas seems to be due to their different rate of thiamine-related glucose and oxidative metabolism (35, 36). This is explained in detail in Supplementary material B.

The three cats from this study with history of epileptic seizures also showed changes at the cerebral cortex, cingulate gyrus and hippocampus. Identical findings have been reported previously in feline patients presenting with history of epileptic seizures (37, 38). It is uncertain whether these changes correspond with primary multifocal parenchymal abnormalities secondary to the deficiency of thiamine or if they are seizure-induced changes similar to those previously described in cats (39, 40). Differentiation between these two conditions would require follow-up MRI and close correlation with the clinical signs and clinical evolution.

### Hypertensive encephalopathy

Magnetic resonance imaging findings of hypertensive encephalopathy were characterised by bilateral and symmetrical ill-defined diffuse lesion of the white matter of the forebrain in all cases (5/5). In agreement with previous studies, these changes were more marked at the parietal to occipital lobes, and could be accompanied by changes at the grey matter structures (such as thalamus and caudate nuclei) (26, 41, 42). When DWI and ADC map were available, the lesions showed facilitated diffusion, suggestive of vasogenic oedema. In humans, hypertensive encephalopathy is considered part of the posterior reversible encephalopathy syndrome (PRES) given the predominant distribution of the vasogenic oedema at the parietal and occipital lobes (43, 44). Interestingly, in this study, cats presented with a more dramatic distribution of the changes, extending from the frontal to the occipital lobes, and with associated mass effect and increased brain volume. An explanation for these differences could be the different vascularization of the feline brain in comparison to dogs. Further detailed discussion about imaging findings and pathophysiology is described in Supplementary material B.

### Exogenous toxins

Several exogenous toxins can be neurotoxic, and in this study, only four different intoxications could be included. Three of five dogs with metronidazole intoxication in our study had morphologically normal brains, whilst the other two dogs showed involvement of multiple grey matter structures, including the red nuclei, vestibular nuclei and cerebellar nuclei. Involvement of the cerebellar nuclei has been previously reported (45, 46). However, lesions affecting the red and vestibular nuclei have only been described in humans (47). Three other types of intoxications included in the study were *Cycas revoluta*, ethylene glycol and bromethalin intoxications. In contrast to metronidazole intoxication, these conditions presented with an increase brain volume and a predominant distribution of the lesions along the white matter. Occasional grey matter involvement (i.e., thalamus) was also seen with *Cycas revoluta* and ethylene glycol intoxications. The imaging findings correlate well with previous published literature (24, 48). A single case report of ethylene glycol toxicity in a dog, described the presence of pachymeningeal enhancement as the only imaging finding (49). It is possible that the pathological changes in this patient were not severe enough to cause visible changes as in our case. In humans with ethylene glycol intoxication describe involvement of thalamus, basal nuclei, amygdala, hippocampus and white matter tracts with associated restricted diffusion (50). The addition of DWI/ADC map sequence in cases with suspicion of intoxication may be useful in order to differentiate between vasogenic and cytotoxic oedema. See Supplementary material B for description of pathophysiology.

### Lysosomal storage disease

From the group of lysosomal storage diseases, a total of 12 neuronal ceroid lipofuscinosis, six gangliosidosis and one dog with fucosidosis were included in our study.

All patients with NCL showed severe brain atrophy resulting from the abnormal accumulation of ceroid- or lipofuscine-like lipopigments as per previous literature descriptions (20, 51–53). Diffuse pachymeningeal enhancement has been previously described in dogs and cats with lipofuscinosis; however, it has not been found in people (20, 53). Amongst the 11 dogs included in this study with NCL, only two showed meningeal contrast enhancement. The reason why meningeal enhancement can be seen in some patients and not in others is unknown, but an autoimmune cause has been alluded in the past (20). It is possible that differences in age, breeds or stages of the disease could trigger the immune response of the patients in different manners leading to the presence/absence of meningeal changes. One of the cases with meningeal contrast enhancement had also a chronic subdural haematoma, similar to the one described in a previous case report (54). Further detailed discussion about imaging findings and pathophysiology is described in Supplementary material B.

The pathophysiology of gangliosidosis and fucosidosis is similar, with the specific substrate accumulation causing gliosis and loss of myelin and neurons (11, 55). On MRI, both conditions also showed similar imaging features characterised by increase in T2W signal intensity of the white matter diffusely and more marked at the internal capsule and corona radiata. These changes are in line with previous veterinary literature (13, 56–58). It is reported that the T2W hyperintensity in the white matter may result from hypoplasia of myelin (hypomyelination) and/or delayed myelination (dysmyelination), increased storage materials and astrocytosis (12). Normal myelination of the white matter is complete at 3–4 months of age, which is an useful information during the diagnosis of these conditions in patients presenting at very young age (59). Contrary to what we observed in our study, corpus callosum abnormalities (and especially hypoplasia) have been reported in animals with juveline-onset gangliosidosis and used as an imaging indicator for this disease (58). However, the cases with both gangliosidosis and fucosidosis in this study showed a normally developed corpus callosum but with increased signal intensity becoming isointense to the grey matter. In humans, a discriminative feature to distinguish fucosidosis from gangliosidosis is the T2W hypointensity of the globus pallidus seen in fucosidosis (7). However, this finding was not present in our case of fucosidosis, or in the single case report of a cat with MRI findings available in the literature (13). It is difficult to stablish specific imaging features of lysosomal storage diseases with the small sample of cases available. There are also many other lysosomal storage diseases not included in our study due to the lack of cases obtained. This in part reflects the low prevalence of these conditions and the difficulty to reach a definitive diagnosis. Future prospective studies with larger samples are needed to analyse these conditions in-depth, being aware that the clinician should still always rely on histopathology or molecular genetic testing available for each lysosomal storage disease in order to reach a definitive diagnosis.

### L-2-hydroxyglutaric aciduria

The 14 cases diagnosed with L-2-hydroxyglutaric aciduria showed a characteristic thickening and T2W and FLAIR hyperintensity of the cortical grey matter along with the involvement of multiple deep grey matter structures. The white matter was affected to a lesser extent, predominantly the subcortical white matter of the forebrain and cerebellar white matter surrounding the cerebellar nuclei (5/14). These imaging findings are in agreement with results from previous literature (23, 60–62). In the single case that DWI and ADC mapping were available, restricted diffusion was present, confirming the cytotoxic origin of the oedema. See Supplementary material B for description of pathophysiology.

### Lafora disease

Lafora disease is characterised by abnormal accumulation of polyglucosan inclusions (also called Lafora bodies) in neurons of all brain regions, reaching the highest density in the substantia nigra followed by the dentate nucleus and thalamic nuclei (63, 64). Contrary to this, none of the 11 dogs with confirmed Lafora disease showed signal intensity changes at these locations. The MRI studies showed either normal brain morphology (5/11) or mild degree of brain atrophy (6/11), which is in agreement with previous human and veterinary literature (10, 63). The interpretation of brain atrophy is complicated given the fact that this imaging finding could be part of the normal ageing process of older dogs (65). The median age of the dogs with Lafora disease in our study was higher than expected for a disease caused by an underlying genetic defect (8.5 years), but typical for the time span reported for the manifestation of Lafora disease in dogs (10). Therefore, it is impossible to definitively determine whether the brain atrophy observed in patients with Lafora disease are caused by the disease itself, by the normal ageing process of the patients or a combination of the two. Further detailed discussion about imaging findings and pathophysiology is described in Supplementary material B.

### Spongiform leukoencephalomyelopathy

A single canine patient was included in the study. The MRI showed bilateral and symmetric, T2W and FLAIR hyperintense lesions affecting the cerebellar nuclei. These lesions were corroborated on histopathology, however, additional spongiosis of the white matter of the forebrain and cerebellum was also identified. This is similar to a case report of a Labrador Retriever diagnosed with spongy degeneration in which MRI showed lesions at the cerebellar nuclei and thalamus, but histopathology also revealed decreased myelin in the white matter (25). In contrast, feline patients have variable MRI findings, with case reports describing lesion with a pure white matter distribution (66), or predominantly affecting grey matter structures (67). The single case of this study is unable to fully represent the imaging features of this disease and future studies with a larger number of cases are needed in order to stablish a pattern recognition for this condition.

### Cerebellar cortical degeneration

Four dogs and two cats were included in the study. In agreement with the previous literature (14, 15, 66), all patients showed atrophy of the cerebellum which can be attributed to the premature loss and degeneration of Purkinje cells identified in histopathology (14). Interestingly, the severity of cerebellar atrophy was more conspicuous in dogs in comparison to cats. This condition is extremely rare in cats and there is no description of the MRI findings in the few case reports that are available in the literature (68–70). The neurological signs in cats typically have a late onset and progress slowly over at least 2 years when compared to dogs (68, 70). In our study, the feline patients were considerably younger than the dogs, with a median age of 6 months compared to 5 years old. It is therefore possible that the clinical presentation at an earlier stage and the naturally slower progression of the disease in cats may explain the different imaging findings between species in this study. No gross differences were seen regarding severity of neurological signs or changes in histopathology between dogs and cats. However, detailed comparison of these features was beyond the scope of the study, and future studies with clinical and histopathological correlation are required.

Evaluation of the brain volume and regions of the brain affected were the most useful factors in developing an MRI pattern recognition system. Metabolic diseases characterised by an increased brain volume included intoxications (with the exception of metronidazole intoxication), L-2-hydroxyglutaric aciduria and hypertensive encephalopathy in cats. Conditions showing a decreased forebrain volume were neuronal ceroid lipofuscinosis (severe brain atrophy), hepatic encephalopathy and Lafora disease (ranging from moderate to mild atrophy). However, it is important to highlight that hepatic encephalopathy and Lafora disease could also present with normal brain volumes. Finally, the only category of disease showing an isolated decrease in cerebellar volume, with normal forebrain volume, was cerebellar cortical degeneration. Regarding the regions of the brain affected, thiamine deficiency and metronidazole intoxication affected exclusively the grey matter structures. Diseases affecting only the white matter included bromethalin intoxication and lysosomal storage diseases (neuronal ceroid lipofuscinosis, gangliosidosis and fucosidosis). The remaining conditions affected both the grey and white matter, however, this was noted on a different extend and a classification was established according to the predominant anatomic distribution. L-2-hydroxyglutaric aciduria and canine spongiform leukoencephalopathy affected predominantly the grey matter, hypertensive encephalopathy and intoxications (Cycad revoluta and ethylene glycol intoxications) had a predominant involvement of the white matter, and hepatic encephalopathy and osmotic demyelination syndrome showed equal involvement of the grey and white matter.

The main limitations of this study are related to its retrospective nature and, despite involvement of 17 different referral institutions, the small case numbers for some of the metabolic encephalopathies in which specific imaging findings may not be completely representative for the overall population. However, this reflects the uncommon occurrence and/or identification of these conditions on the daily basis. In addition, histopathology was not available for all the cases, which would have been helpful to corroborate the imaging findings and especially the novel anatomical locations encountered in some conditions that were not previously described (e.g., cerebellar nuclei with hepatic encephalopathy, the superior longitudinal fasciculus with myelinolysis, etc.). Equally, determination of the genetic variants was not available for all the neurodegenerative cases, which could have been proved useful in the future for new emerging breeds in which a genetic test is not available. Finally, it is important to realise that these MRI guidelines may have exceptions, as the MRI features characteristic for a specific disorder may not be invariably present in all patients or in all stages of the disease.

In conclusion, this is the first study gathering a large number of confirmed metabolic and neurodegenerative encephalopathies in dogs and cats, allowing an in-depth review of the different metabolic conditions and their imaging features, providing additional findings to those already published in the veterinary literature. The current study shows the important role of MRI in distinguishing different metabolic disorders according to specific imaging characteristics, guiding the prioritisation of differential diagnosis, which may facilitate the clinical decision making of the most appropriate diagnostic tests to perform in each specific case.

## Data availability statement

The original contributions presented in the study are included in the article/Supplementary material; further inquiries can be directed to the corresponding author.

## Ethics statement

The animal studies were approved by Professor David Morton CBE MRCVS Chair of the RCVS Ethics Review Panel. The studies were conducted in accordance with the local legislation and institutional requirements. Written informed consent was obtained from the owners for the participation of their animals in this study.

## Author contributions

MM-G: Conceptualization, Investigation, Methodology, Writing – original draft, Writing – review & editing. RG: Resources, Supervision, Writing – review & editing. RQ: Resources, Supervision, Writing – review & editing. PÁ: Resources, Supervision, Writing – review & editing. KB: Resources, Supervision, Writing – review & editing. EA: Resources, Supervision, Writing – review & editing. MMo: Resources, Supervision, Writing – review & editing. EI: Resources, Supervision, Validation, Writing – review & editing. MMa: Resources, Supervision, Writing – review & editing. SG: Resources, Supervision, Writing – review & editing. EG: Resources, Supervision, Writing – review & editing. TB: Resources, Supervision, Writing – review & editing. KS: Resources, Supervision, Writing – review & editing. AV: Resources, Supervision, Writing – review & editing. CB: Resources, Supervision, Writing – review & editing. PM: Resources, Supervision, Writing – review & editing. UI: Resources, Supervision, Writing – review & editing. IC: Conceptualization, Investigation, Methodology, Supervision, Writing – original draft, Writing – review & editing.
