# Correction: Magnetic resonance imaging pattern recognition of metabolic and neurodegenerative encephalopathies in dogs and cats

**DOI:** 10.3389/fvets.2025.1753492

**Published:** 2026-01-09

**Authors:** María Miguel-Garcés, Rita Gonçalves, Rodrigo Quintana, Patricia Álvarez, Katrin M. Beckmann, Emili Alcoverro, Melania Moioli, Edward J. Ives, Megan Madden, Sergio A. Gomes, Evelyn Galban, Tim Bentley, Koen M. Santifort, An Vanhaesebrouck, Chiara Briola, Patricia Montoliu, Unai Ibaseta, Inés Carrera

**Affiliations:** 1 Diagnostic Imaging Department, Southern Counties Veterinary Specialists, Independent Vetcare (IVC) Evidensia, Ringwood, United Kingdom; 2 Department of Small Animal Clinical Science, Small Animal Teaching Hospital, University of Liverpool, Neston, United Kingdom; 3 Small Animal Hospital, School of Biodiversity, One Health and Veterinary Medicine, University of Glasgow, Glasgow, United Kingdom; 4 Neurology and Neurosurgery Department, Pride Veterinary Referrals, Independent Vetcare (IVC) Ltd., Derby, United Kingdom; 5 Section of Neurology, Department of Small Animals, Vetsuisse Faculty Zurich, University of Zurich, Zurich, Switzerland; 6 ChesterGates Veterinary Specialists, Chester, United Kingdom; 7 Division of Clinical Radiology, Department of Clinical Veterinary Medicine, Vetsuisse Faculty, University of Bern, Bern, Switzerland; 8 Anderson Moores Veterinary Specialists, Linnaeus Veterinary Ltd., Hursley, United Kingdom; 9 Royal (Dick) School of Veterinary Studies, University of Edinburgh, Edinburgh, United Kingdom; 10 Dovecote Veterinary Hospital, Castle Donington, United Kingdom; 11 Neurology and Neurosurgery Department, University of Pennsylvania School of Veterinary Medicine, Philadelphia, PA, United States; 12 IVC Evidensia Small Animal Referral Hospital Arnhem, Neurology, Arnhem, Netherlands; 13 IVC Evidensia Small Animal Referral Hospital Hart van Brabant, Neurology, Waalwijk, Netherlands; 14 Queen's Veterinary School Hospital, Veterinary Department, University of Cambridge, Cambridge, United Kingdom; 15 Diagnostic Imaging Service, The Ralph Veterinary Referral Centre, Marlow, United Kingdom; 16 VetCT, Cambridge, United Kingdom; 17 Anicura Ars Veterinaria Hospital Veterinari, Barcelona, Spain; 18 Hospital Veterinari Costa Brava, Girona, Spain; 19 Neurology and Neurosurgery Department, Hospital Veterinario Menes, Gijón, Asturias, Spain; 20 VetOracle, Norfolk, United Kingdom

**Keywords:** metabolic encephalopathies, neurodegenerative encephalopathies, magnetic resonance imaging, grey matter, white matter, MRI recognition pattern

In the published article, an error occurred in the description of the neuroanatomical structure affected in dogs with myelinolysis. The superior longitudinal fasciculus was incorrectly cited as the affected structure and was described as a novel finding. This error appears in the abstract, the **Results** section, Figure 3, and the **Discussion**.

The correct structure involved in dogs with myelinolysis is the claustrum, a grey matter structure located immediately dorsal to the longitudinal fasciculus.

There was a mistake in the caption of Figure 3 as published. The superior longitudinal fasciculus was incorrectly cited as the affected structure: “Dorsal (A) and transverse plane T2-weighted images at the level of the thalamus (B, C) of a dog with presumptive diagnosis of osmotic demyelination syndrome. There are bilateral and symmetric hyperintensities affecting the thalamus (long white arrows) (A–C) and extending into the subthalamus (dashed long white arrows) (C). A specific white matter region located immediately lateral to the internal capsule is also affected, corresponding with the superior longitudinal fasciculus (short black arrows) (B).”

The corrected caption of Figure 3 appears below.

“Dorsal (A) and transverse plane T2-weighted images at the level of the thalamus (B, C) of a dog with presumptive diagnosis of osmotic demyelination syndrome. There are bilateral and symmetric hyperintensities affecting the thalamus (long white arrows) (A–C) and extending into the subthalamus (dashed long white arrows) (C). A specific region of the grey matter is also affected, corresponding with the claustrum (short black arrows) (B).”

In the **Abstract**, the superior longitudinal fasciculus was incorrectly cited as the affected structure and was described as a novel finding: “Metabolic/neurodegenerative encephalopathies encompass a wide list of conditions that share similar clinical and magnetic resonance imaging (MRI) characteristics, challenging the diagnostic process and resulting in numerous tests performed in order to reach a definitive diagnosis. The aims of this multicentric, retrospective and descriptive study are: (I) to describe the MRI features of dogs and cats with metabolic/neurodegenerative encephalopathies; (II) to attempt an MRI recognition pattern classifying these conditions according to the involvement of grey matter, white matter or both; and (III) to correlate the MRI findings with previous literature. A total of 100 cases were recruited, comprising 81 dogs and 19 cats. These included hepatic encephalopathy (20 dogs and three cats), myelinolysis (five dogs), intoxications (seven dogs and one cat), thiamine deficiency (two dogs and seven cats), hypertensive encephalopathy (three dogs and two cats), neuronal ceroid lipofuscinosis (11 dogs and one cat), gangliosidosis (three dogs and two cats), fucosidosis (one dog), L-2-hydroxyglutaric aciduria (13 dogs and one cat), Lafora disease (11 dogs), spongiform leukoencephalomyelopathy (one dog) and cerebellar cortical degeneration (four dogs and two cats). None of the hepatic encephalopathies showed the previously described T1-weighted hyperintensity of the lentiform nuclei. Instead, there was involvement of the cerebellar nuclei (8/23), which is a feature not previously described. Dogs with myelinolysis showed novel involvement of a specific white matter structure, the superior longitudinal fasciculus (5/5). Thiamine deficiency affected numerous deep grey nuclei with novel involvement of the oculomotor nuclei (3/9), thalamic nuclei, subthalamus and cerebellar nuclei (1/9). Cats with hypertensive encephalopathy had a more extensive distribution of the white matter changes when compared to dogs, extending from the parietal and occipital lobes into the frontal lobes with associated mass effect and increased brain volume. Lysosomal storage disease showed white matter involvement only, with neuronal ceroid lipofuscinosis characterised by severe brain atrophy when compared to gangliosidosis and fucosidosis. All patients with L-2-hydroxyglutaric aciduria had a characteristic T2-weighted hyperintense swelling of the cerebral and cerebellar cortical grey matter, resulting in increased brain volume. Lafora disease cases showed either normal brain morphology (5/11) or mild brain atrophy (6/11). Dogs with cerebellar cortical degeneration had more marked cerebellar atrophy when compared to cats. This study shows the important role of MRI in distinguishing different metabolic/neurodegenerative encephalopathies according to specific imaging characteristics.”

This has been corrected to read:

“Metabolic/neurodegenerative encephalopathies encompass a wide list of conditions that share similar clinical and magnetic resonance imaging (MRI) characteristics, challenging the diagnostic process and resulting in numerous tests performed in order to reach a definitive diagnosis. The aims of this multicentric, retrospective and descriptive study are: (I) to describe the MRI features of dogs and cats with metabolic/neurodegenerative encephalopathies; (II) to attempt an MRI recognition pattern classifying these conditions according to the involvement of grey matter, white matter or both; and (III) to correlate the MRI findings with previous literature. A total of 100 cases were recruited, comprising 81 dogs and 19 cats. These included hepatic encephalopathy (20 dogs and three cats), myelinolysis (five dogs), intoxications (seven dogs and one cat), thiamine deficiency (two dogs and seven cats), hypertensive encephalopathy (three dogs and two cats), neuronal ceroid lipofuscinosis (11 dogs and one cat), gangliosidosis (three dogs and two cats), fucosidosis (one dog), L-2-hydroxyglutaric aciduria (13 dogs and one cat), Lafora disease (11 dogs), spongiform leukoencephalomyelopathy (one dog) and cerebellar cortical degeneration (four dogs and two cats). None of the hepatic encephalopathies showed the previously described T1-weighted hyperintensity of the lentiform nuclei. Instead, there was involvement of the cerebellar nuclei (8/23), which is a feature not previously described. Dogs with myelinolysis showed novel involvement of a specific grey matter structure, the claustrum (5/5). Thiamine deficiency affected numerous deep grey nuclei with novel involvement of the oculomotor nuclei (3/9), thalamic nuclei, subthalamus and cerebellar nuclei (1/9). Cats with hypertensive encephalopathy had a more extensive distribution of the white matter changes when compared to dogs, extending from the parietal and occipital lobes into the frontal lobes with associated mass effect and increased brain volume. Lysosomal storage disease showed white matter involvement only, with neuronal ceroid lipofuscinosis characterised by severe brain atrophy when compared to gangliosidosis and fucosidosis. All patients with L-2-hydroxyglutaric aciduria had a characteristic T2-weighted hyperintense swelling of the cerebral and cerebellar cortical grey matter, resulting in increased brain volume. Lafora disease cases showed either normal brain morphology (5/11) or mild brain atrophy (6/11). Dogs with cerebellar cortical degeneration had more marked cerebellar atrophy when compared to cats. This study shows the important role of MRI in distinguishing different metabolic/neurodegenerative encephalopathies according to specific imaging characteristics.”

The superior longitudinal fasciculus was incorrectly cited as the affected structure and was described as a novel finding. This error appears in the Results section and the Discussion. The correct structure involved in dogs with myelinolysis is the claustrum.

A correction has been made to the section **Results**, subsection *endogenous malfunction and toxins—Myelinolysis/osmotic demyelination syndrome*, Paragraph Number 9: “The brain volume was normal in all cases (5/5). Patients showed both grey and white matter structures affected with the presence of bilateral, symmetric intra-axial lesions. The grey matter structures affected included thalamus (5/5) and subthalamus (1/5). Regarding the white matter, a specific anatomical region located immediately lateral to the internal capsule was affected in all cases (5/5), corresponding with the superior longitudinal fasciculus (Figure 3). All lesions were homogenous, hyperintense in T2W and FLAIR, isointense in T1W and without contrast *enhancement.”*

The correct statement is: “The brain volume was normal in all cases (5/5). Patients showed grey matter structures affected with the presence of bilateral, symmetric intra-axial lesions. The grey matter structures affected included thalamus (5/5), subthalamus (1/5) and a specific anatomical region located immediately lateral to the internal capsule (5/5), corresponding with the claustrum (Figure 3).”

A correction has been made to the section **Discussion**, subsection *Osmotic demyelination syndrome*, Paragraph Number 5: “A novel finding not previously described was the involvement of a specific white matter structure seen in all patients, the superior longitudinal fasciculus. The superior longitudinal fasciculus is a type of long association fibre contained within the white matter that connects the frontal cortex with the parietal and the occipital cortex (27). The reason for the susceptibility of this specific region in these patients is unknown and histopathological correlation is required to confirm the nature of the changes.”

The correct statement is: “A novel finding not previously described was the involvement of a specific grey matter structure seen in all patients, the claustrum. The claustrum is a component of the basal nuclei (27). The reason for the susceptibility of this specific region in these patients is unknown and histopathological correlation is required to confirm the nature of the changes.”

The original version of this article has been updated.

