# Supplementary material for: Magnetic resonance imaging pattern recognition of metabolic and neurodegenerative encephalopathies in dogs and cats
Source: Front Vet Sci. 2024 Jul 30;11:1390971. doi: 10.3389/fvets.2024.1390971 (PMC11319130; doi:10.3389/fvets.2024.1390971)
Supplement: Supplementary file 1 [file Table_1.docx]

Supplementary Material

**Magnetic resonance imaging pattern recognition of metabolic and neurodegenerative encephalopathies in dogs and cats**

María Miguel-Garcés^1*^ †, Rita Gonçalves^2^, Rodrigo Quintana^3^, Patricia Álvarez^4^, Katrin M. Beckmann^5^, Emili Alcoverro^6^, Melania Moioli^7^, Edward J. Ives^8^, Megan Madden^9^, Sergio A. Gomes^10^, Evelyn Galban^11^, Tim Bentley^2^, Koen M. Santifort^12^, An Vanhaesebrouk^13^, Chiara Briola^14^, Patricia Montoliu^15^, Unai Ibaseta^16^, Inés Carrera^17^ †.

***Correspondence:**

María Miguel-Garcés

[miguelgarcesmaria@gmail.com](mailto:miguelgarcesmaria@gmail.com)

# Supplementary Data: Results.

- 1. 1. Hepatic encephalopathy
- Dogs
- Breakdown of breeds and number of cases: Shih Tzu (n= 4), Border Terrier (n= 2), Standard Schnauzer (n=2), West Highland White Terrier (n= 2), Cairn Terrier (n= 1), Chihuahua (n= 1), English Cocker Spaniel (n= 1), English Sheepdog (n= 1), Entlebucher Mountain dog (n = 1), Lurcher (n= 1), Miniature Pinscher (n= 1), Labradoodle (n= 1), Poodle (toy) (n= 1) and a crossbreed (n = 1).
- Neurological signs: Obtundation (n= 12), proprioceptive deficits (n= 6), ataxia (n= 5), pacing/circling (n= 7), reduced menace response (n= 7), abnormal behaviour (n= 6), epileptic seizures (n= 4), head pressing (n= 2) and non-ambulatory tetraparesis (n=1).
- Inclusion criteria (diagnosis of hepatic encephalopathy, treatment and outcome): In two dogs the cause was the presence of multiple acquired shunts secondary to advanced liver disease (liver cirrhosis), whether the remaining patients presented with congenital portosystemic shunts of variable morphology. The diagnosis was made by a combination of elevated pre- and post-prandial bile acids (n= 18) and confirmation of portosystemic shunts by ultrasound (n= 10) or computed tomography (n= 8). Necropsy/histopathology was performed in 3 cases. Surgical attenuation of the shunt was performed in 6 patients, whilst 12 were treated medically. Follow-up was available only in 11 cases; there was a good outcome reported in 7 cases, whereas the other 4 cases were euthanised due to deterioration of the neurological signs.
- Other laboratory findings: Elevation of alanine aminotransferase (ALT) (n= 14), hypoalbuminemia (n= 8), microcytosis (n= 3) and ammonia (n= 11 patients).
- Cats
- Breakdown of breeds and number of cases: Domestic longhair (n= 1), British Shorthair (n= 1) and Sphynx (n= 1).
- Neurological signs: proprioceptive deficits (n= 3), ataxia (n= 3), obtundation (n= 1) and non-ambulatory tetraparesis (n= 1).
- Inclusion criteria (diagnosis of hepatic encephalopathy, treatment and outcome): The diagnosis was made by a combination elevated pre- and post-prandial bile acids (n=3) and confirmation of portosystemic shunts by ultrasound (n= 1) or computed tomography (n= 2). All patients were treated medically. Follow-up was not available for any of the cases.
- Other laboratory findings: Elevation of ALT (n= 2) and ammonia (n= 1).
  1. 2. Myelinolysis/Osmotic demyelination syndrome
- Breakdown of breeds and number of cases: English Cocker Spaniel (n= 1), Lhasa Apso (n= 1), Standar Schnauzer (n= 1) and crossbreed (n= 2).
- Neurological signs: Obtunded (n= 4), with proprioceptive deficits (n= 3), diminished bilateral menace response (n= 1) and tendency for circling (n= 1). One case (n= 1) had epileptic seizures at presentation, with a reduced level of mentation, an intermittent head tilt and non-ambulatory tetraparesis.
- Inclusion criteria (diagnosis, treatment and outcome): The diagnosis of myelinolysis was assumed in all cases based on the clinical history (neurological deterioration following the rapid correction of hyponatremia) and clinical improvement following symptomatic treatment.
  1. 3. Nutritional imbalance – Thiamine deficiency
- Dogs
- Neurological signs: Ataxia (n= 2), spontaneous nystagmus (n= 2), cervical ventroflexion (n= 1), non-ambulatory tetraparesis (n= 1), reduced menace response (n= 1), decreased pupillary light reflex (n= 1) and proprioceptive deficits (n= 1).
- Inclusion criteria (diagnosis, treatment and outcome): The diagnosis of this condition was made by a combination of patient history (change to nutritionally unbalanced diet), specific clinical signs and evidence of low thiamine levels measured in blood (1/2) and/or good response after thiamine supplementation (2/2). Follow up showed good prognosis in both dogs with resolution of the neurological signs.
- Cats
- Breakdown of breeds and number of cases: Domestic shorthair (n= 5), British Shorthair (n= 1) and Ragdoll (n= 1).
- Neurological signs: Ataxia (n= 6), cervical ventroflexion (n= 4), non-ambulatory tetraparesis (n= 4), reduced menace response (n= 4), epileptic seizures (n= 3), spontaneous nystagmus (n= 1), decreased pupillary light reflex (n= 1), head tremor (n= 2), proprioceptive deficits (n= 1), vision loss (n= 2), head tilt (n= 1), hypermetria (n= 1), mydriasis (n= 1) and strabismus (n= 1).
- Inclusion criteria (diagnosis, treatment and outcome): The diagnosis was made by a combination of patient history, specific clinical signs and evidence of low thiamine levels measured in blood (5/7) or good response after thiamine supplementation (2/7). Follow-up was available in all cases which showed good prognosis with resolution of the neurological signs.
  1. 4. Blood flow disturbance – Hypertensive encephalopathy
- Dogs
- Breakdown of breeds and number of cases: Bedlington Terrier (n= 1), Dalmatian (n= 1) and a crossbreed (n= 1).
- Neurological signs: Epileptic seizures (n= 1), proprioceptive deficits (n= 2), obtundation (n= 1), ataxia (n= 2), generalised tremors (n= 1), hypermetric gait (n= 1), tendency to circle (n= 1) and absent menace response (n= 1).
- Diagnosis: The diagnosis was made based on the confirmation of systemic hypertension (> 160 mmHg), which was secondary to chronic renal disease in two dogs and due to an adrenal gland pheochromocytoma in one dog. Follow-up was not available.
- Cats
- Neurological signs: Epileptic seizures (n= 2), proprioceptive deficits (n= 2), non-ambulatory tetraparesis (n= 2), obtundation (n= 2) and absent menace response (n= 1).
- Diagnosis: Histopathology/necropsy confirmed the diagnosis of hypertensive encephalopathy secondary to chronic renal disease.
  1. 1. Exogeneous toxins
- Dogs
- Breakdown of breeds and number of cases: Labrador Retriever (n= 2), German Shepherd (n= 1), Golden Retriever (n= 1), Jack Russell Terrier (n= 1), Northern Inuit (n= 1) and Siberian Husky (n= 1).
- Neurological signs: Acute history of ataxia (n= 5), nystagmus (n= 5), intermittent opisthotonos (n= 4), obtundation (n= 2), head tilt (n= 3), wide-base stance (n= 3), reduced menace response (n= 2) and intention tremors (n= 1).
- Inclusion criteria (diagnosis, treatment and outcome): The diagnosis was reached by necropsy/histopathology in the case of ethylene glycol intoxication. The diagnosis for all the metronidazole intoxications was made based on the acute signs after implementation of the drug within the therapy and rapid recovery once stopped it. The diagnosis of the *Cycas revoluta* intoxication case was reached by a combination of acute clinical signs, clinical history of access to the plant and improvement following supportive treatment. The prognosis was good for all cases except for the ethylene glycol intoxications in which rapid deterioration led to euthanasia.
- Other laboratory findings: Increased creatinine and blood urea nitrogen (BUN) with presence of hyperphosphatemia for the dog with ethylene glycol intoxication.
- Cat
- Neurological signs: Obtundation, nystagmus and proprioceptive deficits in all limbs.
- Diagnosis: The diagnosis was reached by necropsy/histopathology.
  1. 1. Lysosomal storage diseases

1.a Neuronal ceroid lipofuscinosis

- Dogs
- Breakdown of breeds and number of cases: Chihuahua (n= 6), Field Spaniel (n= 1), Jack Russell (n= 1), Tibetan Terrier (n= 1) and crossbreed (n=2).
- Neurological signs: Decreased to absent menace response (n= 10), ataxia (n= 9), behavioural abnormalities (n= 9), proprioceptive deficits (n= 5), head tilt (n= 4), intention tremors (n= 3) and obtundation (n= 2).
- Inclusion criteria (diagnosis and outcome): The diagnosis of the condition was obtained by a specific genetic test for the Chihuahua breed - *MFSD8* gene [c. 843deIT] (n= 6) or necropsy/histopathology including fluorescence and electron micrographs which showed intracytoplasmic accumulation of autofluorescent lysosomal storage bodies in many neurons as the main characteristic finding (n= 5). Follow-up was available in 6 cases in which the prognosis was poor with all the patients showing progressive deterioration of the clinical signs leading to euthanasia.
- Cat
- Neurological signs: One isolated seizure, intermittent opisthotonos, obtundation and reduced menace response.
- Diagnosis: Necropsy/histopathology including fluorescence and electron micrographs which showed the intracytoplasmic accumulation of autofluorescent lysosomal storage bodies in many neurons.

1.b GM1 and GM2 gangliosidosis

- Dogs
- Neurological signs: Progressive cerebellar ataxia (n=3), positional nystagmus (n= 2), proprioceptive deficits (n= 1) and absent menace response (n= 1).
- Diagnosis: The diagnosis was obtained by a genetic test for the *HEXB* gene [c.618-620delCCT] (1/3), necropsy/histopathology (1/3) or a combination of both (1/3).
- Cats
- Neurological signs: Progressive cerebellar ataxia (n= 2), positional nystagmus (n= 1), absent menace response (n= 1), intention tremors (n= 1) and tonic-clonic seizures (n =1).
- Diagnosis: Necropsy/histopathology was performed in both cats.

1.c Fucosidosis

- Dog
- Neurological signs: Apprehensive behaviour, cerebellar ataxia with hypermetric gait, generalised tremors and positional nystagmus.
- Diagnosis: The diagnosis was made by a genetic test for the FUCA1 gene.

1.3 2. L-2-hydroxyglutaric aciduria

- Dogs
- Breakdown of breeds and number of cases: Staffordshire Bull Terrier (n= 9), Yorkshire (n= 1), West Highland White Terrier (n= 1) and crossbreed (n= 2).
- Neurological signs: Ataxia (n= 8), hypermetria (n= 8), behavioural changes (n= 6), stiffness/muscle cramping of four limbs (n= 4), decreased menace response (n= 2), proprioceptive deficits (n= 3), epileptic seizures (n= 2), generalised tremors (n= 2), kyphosis (n= 2), wide-based posture (n= 2) and head pressing (n= 1). In three patients the neurological signs were more accentuated after exercise or excitement.
- Diagnosis: The disease was diagnosed by urinary organic acid screening (2/13), genetic test of the *L2HGDH* [c.1A>G] and *L2HGDH* [c.1298_1300delinsCTT] genes (5/13) or both (6/13).
- Cat
- Neurological signs: Behavioural changes, circling and decreased menace response.
- Diagnosis: Urinary organic acid screening.

1.3 3. Lafora disease

- Dogs
- Breakdown of breeds and number of cases: Beagle (n= 5), Basset hound (n= 2), Brussels Griffon (n= 1), Chihuahua (n =1), Wire haired Dachshund (n= 1) and crossbreed (n= 1).
- Neurological signs: Spontaneous and myoclonic events triggered by noises or visual stimuli (n=4), generalised tonic-clonic seizures (n=3), or a combination of both (n=4). Additional neurological signs included cerebellar ataxia (n=2), intention tremors (n=1) and visual deficits (n=1).
- Inclusion criteria (diagnosis, treatment and outcome): Lafora disease was confirmed by a genetic test for the NHLRC1 gene variant in all dogs. The outcome was available only in 4 cases in which the neurological signs progressed over the next year but there was a good control of myoclonic and epileptic events with antiseizure medication.

1.3 4. Spongiform leukoencephalomyelopathy

- Neurological signs: Ataxia, intention tremors, hypermetria and proprioceptive deficits.

- Diagnosis: Histopathology/necropsy.

1.3 5. Cerebellar cortical degeneration

- Dogs
- Breakdown of breeds and number of cases: German Wire Hired Pointer (n= 1), Jack Russell (n= 1), Shiba Inu (n= 1) and Staffordshire Bull terrier (n= 1).
- Neurological signs: Cerebellar ataxia (n=4), intention tremors (n=1) and absent menace response (n=2). All patients were normal at birth and developed neurological signs between 2 to 32 weeks prior presentation.
- Diagnosis: Necropsy/histopathology in all cases.
- Cats
- Neurological signs: Cerebellar ataxia (n=2) and intention tremors (n=2).
- Diagnosis: Necropsy/histopathology in both cases.
