# Supplementary material for: Magnetic resonance imaging pattern recognition of metabolic and neurodegenerative encephalopathies in dogs and cats
Source: Front Vet Sci. 2024 Jul 30;11:1390971. doi: 10.3389/fvets.2024.1390971 (PMC11319130; doi:10.3389/fvets.2024.1390971)
Supplement: Supplementary file 2 [file Table_2.docx]

Supplementary Material

**Magnetic resonance imaging pattern recognition of metabolic and neurodegenerative encephalopathies in dogs and cats**

María Miguel-Garcés^1*^ †, Rita Gonçalves^2^, Rodrigo Quintana^3^, Patricia Álvarez^4^, Katrin M. Beckmann^5^, Emili Alcoverro^6^, Melania Moioli^7^, Edward J. Ives^8^, Megan Madden^9^, Sergio A. Gomes^10^, Evelyn Galban^11^, Tim Bentley^2^, Koen M. Santifort^12^, An Vanhaesebrouk^13^, Chiara Briola^14^, Patricia Montoliu^15^, Unai Ibaseta^16^, Inés Carrera^17^ †.

***Correspondence:**

María Miguel-Garcés

[miguelgarcesmaria@gmail.com](mailto:miguelgarcesmaria@gmail.com)

# Supplementary Data: Discussion.

*Hepatic encephalopathy*

The MRI findings suggestive of oedema can be explained by the complex pathogenesis of hepatic encephalopathy in which increased concentration of ammonia crosses the blood-brain barrier, and via the glutamate-glutamine cycle the ammonia gets converted into glutamine. Excessive accumulation of glutamine within the astrocyte leads to osmotic stress, astrocyte swelling, and finally brain oedema (1).

Contrary to previous publications (2, 3), none of our cases showed abnormal increased of T1W signal intensity of the lentiform nuclei. The lentiform nucleus is composed of two parts, the putamen and the globus pallidus, which contain high amount of myelin fibers forming the internal connections of the basal ganglia (4). On MRI the increase in myelin content will cause shortening of the T1 relaxation with subsequent increase in the hyperintensity of this region on conventional T1W images. It is then suspected that the inherent mild hyperintense appearance of this region on T1W images of normal dogs is commonly overestimated.

*Osmotic demyelination syndrome*

Osmotic demyelination syndrome occurs due to a depleted adaptive process to protect against brain swelling, in which the redistribution of solutes with correction of hyponatremia causes brain shrinkage, leading to disruption of tight junctions and disruption of the blood-brain barrier, causing oligodentrocyte damage and neuronal demyelination (5). These explains the appearance of the lesions in MRI. However, it is still unclear why certain areas of the brain, such as the pons, are more susceptible to myelin damage (6).

*Thiamine deficiency*

Thiamine is an essential cofactor in the glucose metabolism and energy production. Tissues with higher requirement of glucose as the primary source of energy are more affected by thiamine deficiency than are other tissues in the body that have the ability to use lactate or pyruvate for energy. This explains why certain areas in the brain are more affected by thiamine deficiency than others (7,8).

*Hypertensive encephalopathy*

Dogs and cats show a predominant distribution of the changes at the parietal and occipital lobes similar to humans. The predilection for the posterior regions of the cerebrum in humans is explained by a difference in the sympathetic innervation of the cerebral vasculature in which the degree of sympathetic innervation decreases from anterior to posterior regions of the brain, with the least present at the basilar artery. As a result, the more sparsely innervated vascular beds of the posterior regions of the cerebrum may be more susceptible to the effects of hypertension (9). It would be reasonable to assume that a similar theory could be applied to our veterinary patients.

On the other hand, cats presented a more dramatic distribution of the changes, being more extensive and with associated mass effect. This could be explained by the different vascularization of the feline and canine brains. Feline cerebral vascular supply is mainly by the maxillary artery (after branching from external carotid artery), while canine cerebral perfusion is supplied by the basilar and internal carotid arteries. In addition, the arterial circle in the cat is not a closed ring because of the lack of a rostral communicating artery that is normally present in other species, and the direction of flow in the basilar artery is away from the arterial circle. This means that maxillary blood from the external carotid arteries is distributed to the entire brain, except for the caudal portion of the brainstem, which is supplied by the vertebral arteries (10).

*Exogenous toxins*

It is believed that toxins may mimic the effect of glutamate resulting in an excessive excitatory effect in the synaptic cleft, causing a cascade of enzyme activation and leading to neuronal damage due to excitotoxicity (cell swelling and death) (11, 12, 13).

*Lysosomal storage diseases*

- *Neuronal ceroid lipofuscinosis*

The abnormal accumulation of ceroid- or lipofuscin-like lipopigments results in progressive and selective neuronal loss, coupled with astrocytic proliferation and hypertrophy as well as macrophage infiltration (14,15). This neuronal destruction will lead to the characteristic severe brain atrophy encountered in patients suffering from this disease (16, 17).

The progression of the atrophy can cause tearing of blood vessels from the dura and subsequently result in a subdural hematoma formation as the one noted in our case and previous bibliography (18).

Diffuse enhancement of the pachymeninges can also be encountered. Histopathologically, this enhancement can be explained by fibrin deposit and infiltration of macrophages and plasma cells in the meninges, suggesting an inflammatory response to some type of material (16).

*L-2-hydroxyglutaric aciduria*

In the brain, the grey matter is primarily affected, although changes can extend into the adjacent white matter of the cerebellar folia and some regions of the cerebral hemispheres (19). Microscopically, these lesions correspond with spongiform changes, characterised by vacuolar swelling of perivascular and perineuronal astrocytes suggestive of cytotoxic oedema (20). The exact pathogenesis is unknown, but it may represent a toxic effect of the L-2-hydroxyglutaric acid accumulation which is demonstrated to be mediated through induction of oxidative stress and mitochondrial dysfunction (21).

*Lafora disease*

The mechanism by which the accumulation of Lafora bodies results in myoclonic epilepsy is not yet completely understood, but there is increasing evidence that the accumulation of glycogen inclusion in astrocytes results in inflammation, astrocyte reactivity and activation of microglia triggering seizure activity (22).

# References.

1. CARRERA, I., KIRCHER, P. R., MEIER, D., RICHTER, H., BECKMAN, K. and DENNLER, M. (2014). In vivo proton magnetic resonance spectroscopy for the evaluation of hepatic encephalopathy in dogs. *American journal of veterinary research,* 75 (9), 818-827. Doi: 10.2460/ajvr.75.9.818

2. TORISU, S., WASHIZU, M., HASEGAWA, D. and ORIMA, H. (2005). Brain magnetic resonance imaging characteristics in dogs and cats with congenital portosystemic shunts. *Veterinary Radiology & Ultrasound,* 46 (6), 447-451. Doi: 10.1111/j.1740-8261.2005.00082.x

3. MORTERA‐BALSA, V., PENDERIS, J., WESSMANN, A., GONÇALVES, R., LOWRIE, M. and GUTIERREZ‐QUINTANA, R. (2015). Magnetic resonance imaging of the lentiform nuclei in dogs with portosystemic shunts. *Journal of Small Animal Practice,* 56 (5), 307-311. Doi: 10.1111/jsap.12337

4. GIL, V.A. (2022). Neuroanatomy of the dog. New York: Linus Learning.

5. ZUNGA, P. M., FAROOQ, O., DAR, M. I., DAR, I. H., RASHID, S., RATHER, A. Q., et al. (2015). Extra pontine osmotic demyelination syndrome. *Annals of neurosciences,* 22 (1), 51.
Doi: 10.5214/ans.0972.7531.220212

6. GENCPINAR, P., TEKGUC, H., SENOL, A. U., DUMAN, O. and DURSUN, O. (2014). Extrapontine myelinolysis in an 18-month-old boy with diabetic ketoacidosis: case report and literature review. *Journal of Child Neurology,* 29(11), 1548-1553. Doi: 10.1177/0883073813520496

7. MARKOVICH, J. E., HEINZE, C. R. and FREEMAN, L. M. (2013). Thiamine deficiency in dogs and cats. *Journal of the American Veterinary Medical Association,* 243(5), 649-656. Doi: 10.2460/javma.243.5.649

8. CHANG, Y.-P., CHIU, P.-Y., LIN, C.-T., LIU, I.-H. and LIU, C.-H. (2017). Outbreak of thiamine deficiency in cats associated with the feeding of defective dry food. *Journal of feline medicine and surgery,* 19 (4), 336-343. Doi: 10.1177/1098612X15625353

9. CHEN, S.-P., FUH, J.-L. and WANG, S.-J. (2010). Reversible cerebral vasoconstriction syndrome: an under-recognized clinical emergency. *Therapeutic advances in neurological disorders,* 3 (3), 161-171. Doi: 10.1177/1756285610361795

10. ALTAY, U. M., SKERRITT, G. C., HILBE, M., EHRENSPERGER, F. and STEFFEN, F. (2011). Feline cerebrovascular disease: clinical and histopathologic findings in 16 cats. *Journal of the American Animal Hospital Association,* 47 (2), 89-97. Doi: 10.5326/JAAHA-MS-5480

11. DE OLIVEIRA, A. M., PAULINO, M. V., VIEIRA, A. P., MCKINNEY, A. M., DA ROCHA, A. J., DOS SANTOS, G. T., et al. (2019). Imaging patterns of toxic and metabolic brain disorders. *Radiographics,* 39 (6), 1672-1695. doi: 10.1148/rg.2019190016

12. MURTHY, V. D., MCLARTY, E., WOOLARD, K. D., PARKER, R. L., KORTZ, G., KING, J. N. et al. (2022). Case report: MRI, clinical, and pathological correlates of bromethalin toxicosis in three dogs. *Frontiers in Veterinary Science,* 9, 879007. Doi: 10.3389/fvets.2022.879007

13. MAESO, C., MORALES, C., OBRADOR, R., ABARCA, E. and CARRERA, I. (2020). Presumptive Cycad Toxicosis in a Dog. Clinical and Magnetic Resonance Imaging Findings: A Case Report. *Frontiers in Veterinary Science,* 7, 468. Doi: 10.3389/fvets.2020.00468

14. Katz, M.L., Rustad, E., Robinson, G.O., Whiting, R.E., Student, J.T., Coates, J.R., et al. (2017). Canine neuronal ceroid lipofuscinoses: Promising models for preclinical testing of therapeutic interventions. *Neurobiology of disease*, 108, 277-87. Doi:10.1016/j.nbd.2017.08.017

15. NELVAGAL, H. R., LANGE, J., TAKAHASHI, K., TARCZYLUK-WELLS, M. A. and COOPER, J. D. (2020). Pathomechanisms in the neuronal ceroid lipofuscinoses. *Biochimica et Biophysica Acta (BBA)-Molecular Basis of Disease,* 1866 (9), 165570. Doi: 10.1016/j.bbadis.2019.165570

16. NAKAMOTO, Y., YAMATO, O., UCHIDA, K., NIBE, K., TAMURA, S., OZAWA, T., et al. (2011). Neuronal ceroid-lipofuscinosis in longhaired Chihuahuas: clinical, pathologic, and MRI findings. *Journal of the American Animal Hospital Association,* 47 (4), e64-e70. Doi: 10.5326/JAAHA-MS- 5564

17. KOIE, H., SHIBUYA, H., SATO, T., SATO, A., NAWA, K., NAWA, Y. et al. (2004). Magnetic resonance imaging of neuronal ceroid lipofuscinosis in a border collie. *Journal of veterinary medical science,* 66 (11), 1453-1456. Doi:10.1292/jvms.66.1453

18. ASAKAWA, M. G., MACKILLOP, E., OLBY, N. J., ROBERTSON, I. D. and CULLEN, J. M. (2010). Imaging diagnosis—Neuronal ceroid lipofuscinosis with a chronic subdural hematoma. *Veterinary Radiology & Ultrasound,* 51 (2), 155-158. Doi: 10.1111/j.1140-8261.2009.01642.x

19. ABRAMSON, C. J., PLATT, S. R., JAKOBS, C., VERHOEVEN, N. M., DENNIS, R., GAROSI, L. et al. (2003). L‐2‐hydroxyglutaric aciduria in Staffordshire bull terriers. *Journal of Veterinary Internal Medicine,* 17 (4), 551-556. Doi: 10.1111/j.1939-1676.2003.tb02477.x

20. SCURRELL, E., DAVIES, E., BAINES, E., CHERUBINI, G., PLATT, S., BLAKEMORE, W., WILLIAMS, A. and SCHÖNIGER, S. (2008). Neuropathological findings in a Staffordshire bull terrier with l-2-hydroxyglutaric aciduria. *Journal of comparative pathology,* 138 (2-3), 160-164. Doi: 10.1016/j.jcpa.2007.11.005

21. PENDERIS, J., CALVIN, J., ABRAMSON, C., JAKOBS, C., PETTITT, L., BINNS, M. M. et al. (2007). L-2-hydroxyglutaric aciduria: characterisation of the molecular defect in a spontaneous canine model. *Journal of medical genetics,* 44 (5), 334-340. Doi: 10.1136/jmg.2006.042507

22. VON KLOPMANN, T., AHONEN, S., ESPADAS-SANTIUSTE, I., MATIASEK, K., SANCHEZ- MASIAN, D., RUPP, S., et al. (2021). Canine Lafora disease: an unstable repeat expansion disorder. *Life,* 11 (7), 689. Doi: 10.3390/life11070689
